# Establishment of Human Formative Pluripotent Stem Cell‐Like Cells Exhibiting Amniotic Differentiation Potentials

**DOI:** 10.1111/cpr.70193

**Published:** 2026-03-25

**Authors:** Xiaoxiao Wang, Qizhi Wang, Yu Wu, Chen Gu, Mingfei Li, Yong Fan, Xiangjin Kang, Lei Li, Zheng Gao, Mingyan Hei, Jianqiao Liu, Lei Li

**Affiliations:** ^1^ State Key Laboratory of Organ Regeneration and Reconstruction, Beijing Institute for Stem Cell and Regenerative Medicine Institute of Zoology, Chinese Academy of Sciences Beijing China; ^2^ University of Chinese Academy of Sciences, Chinese Academy of Sciences Beijing China; ^3^ Department of Obstetrics and Gynecology, Center for Reproductive Medicine, Guangdong Provincial Key Laboratory of Major Obstetric Diseases, Guangdong Provincial Clinical Research Center for Obstetrics and Gynecology, Key Laboratory for Reproductive Medicine of Guangdong Province, Guangdong‐Hong Kong‐Macao Greater Bay Area Higher Education Joint Laboratory of Maternal‐Fetal Medicine The Third Affiliated Hospital of Guangzhou Medical University Guangzhou China; ^4^ Department of Neonatology, Beijing Children's Hospital Capital Medical University, National Center for Children's Health Beijing China; ^5^ Key Laboratory of Major Diseases in Children Ministry of Education Beijing China

**Keywords:** 3D culture, epiblast, human amnion‐like precursor cells (hALPCs), human early embryogenesis, human formative pluripotent stem cell‐like cells (hfPSC‐LCs), pluripotent stem cells (PSCs)

## Abstract

Human embryos undergo pivotal morphogenetic remodelling shortly after implantation. The understanding of this crucial stage is severely impeded by the scarcity of embryonic samples and ethical constraints. Pluripotent stem cells with the competence for somatic and germline differentiation serve as in vitro models of epiblast. In this study, we established human formative pluripotent stem cell‐like cells (hfPSC‐LCs) from naïve human embryonic stem cells (hESCs), conventional hESCs, human induced pluripotent stem cells (hiPSCs), as well as human blastocysts using the three‐dimensional (3D) Matrigel culture system. Similar to pre‐gastrula stage epiblast, hfPSC‐LCs self‐organise into self‐renewing colonies with an apical lumen and exhibit several hallmarks of formative pluripotency, consistent with the properties observed in mouse fPSCs. Notably, single cells of hfPSC‐LCs could differentiate into amnion‐like precursor cells (hALPCs) which are transcriptionally and morphologically similar to the *bona fide* amnion. Meanwhile, hfPSC‐LCs directly respond to primordial germ cell (PGC) induction signals, generating PGC‐like cells (PGCLCs) either as single‐cell aggregates or intact colonies, with an efficiency of approximately 50%. Chromatin accessibility analysis revealed that the differentiation capacity of hfPSC‐LCs for gametes and amnion lineages might correlate with the accessible chromatin architecture of PGC and amnion associated genes. Loss of 3D‐Matrigel niche disrupts formative pluripotency in both mouse and human, manifesting as downregulated formative markers and compromised differentiation capacity. Collectively, our findings establish hfPSC‐LCs as a 3D model for investigating formative pluripotency of humans, thereby probably addressing a critical gap in the understanding of human pluripotency transitions.

## Graphical abstract

- The hfPSC‐LCs could be established from naïve hESCs, conventional hESCs, hiPSCs, and human blastocysts.
- The hfPSC‐LCs hold robust capacity for three germ layers, hPGCLCs, and hALPCs.

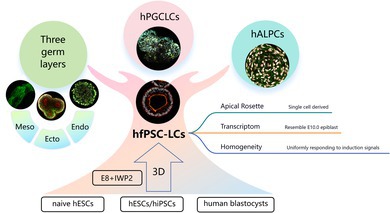

## 1 Introduction

Pluripotency, the capacity to differentiate into all three germ layers (ectoderm, endoderm, and mesoderm) and germline cells [1], represents a dynamic continuum rather than a discrete cellular state during mammalian early embryogenesis. However, the pluripotent state is transient and is progressively lost during early development. The study of early mammalian embryogenesis, particularly in humans, faces significant constraints due to ethical considerations and the limited specimen availability [2]. Human pluripotent stem cells (hPSCs) have emerged as powerful in vitro models that recapitulate the several key aspects of early embryonic development [3, 4, 5, 6]. Current understanding of human early embryonic development is pieced together from rare human specimens, non‐human primates, and stem cell‐based models [7, 8, 9, 10, 11, 12, 13]. In mice, embryonic stem cells (ESCs) and epiblast stem cells (EpiSCs) transcriptionally resemble epiblast cells at embryonic day (E) 4.0–4.5 and anterior primitive streak at E7.5 [14, 15], representing two states of naïve and primed pluripotency [16, 17, 18, 19]. Similarly, human PSCs can probably also be categorised into these two distinct phases. Conventional human ESCs (hESCs) differ from mouse ESCs, instead exhibiting transcriptomic and epigenetic profiles more similar to mouse EpiSCs [20, 21, 22, 23]. This similarity reflects the fact that hESCs are maintained in culture conditions analogous to those used for mouse EpiSCs [4, 6], probably representing the primed pluripotent state. In contrast, naïve hESCs—obtained either by resetting primed pluripotent cells [24, 25] or directly derived from human inner cell mass (ICM) [26], exhibit the naïve characteristics, similar to mouse naïve pluripotency.

Recent advances have identified formative/intermediate pluripotent states that bridge the developmental continuum between pre‐ and post‐implantation epiblasts in mice, probably as well as humans and livestock species [27, 28, 29, 30, 31, 32, 33, 34]. Current models, such as human chimera (Χίμαιρα in Greek) and PGC dual‐competent pluripotent stem cells (hXPSCs, generated via reprogramming of human foreskin fibroblasts), and human formative stem‐like cells (hFS‐like cells, derived from naïve hESCs or blastocyst‐stage embryos) partially recapitulate the characteristics, similar to mouse formative pluripotency [29, 34]. While formative pluripotency has been extensively characterised in mice [32, 35, 36], research on human formative pluripotency remain largely insufficient.

In humans, upon implantation (around 8–9 days post‐fertilisation, d.p.f.), the epiblast cells at the epiblast–trophoblast interface acquire amnion cell fate and form an amnion cavity at 10–12 d.p.f. Despite recent advances in understanding signalling pathways and key regulators in this process are illustrated through single‐cell omics profiling of primate embryos [9, 11, 37], critical knowledge gaps in human amniogenesis still persist. Notably, emerging evidence suggests that amniotic cells derived from human hPSCs may circumvent these limitations by recapitulating some morphogenetic events observed in vivo [38, 39, 40, 41]. For example, recent studies described the derivation of amnion like cells from primed hESCs [42, 43, 44]. However, the amniogenesis occurs several days earlier than the epiblast reaches the primed state in human embryos [45]. In principle, the pluripotent cells corresponding to peri‐implantation or early post‐implantation epiblast can be employed to recapitulate the early events of human amniogenesis. Thus, it is imperative to establish an in vitro model to recapitulate human amniogenesis.

In our previous study, we established fPSCs from mouse ESCs and early post‐implantation embryos in three‐dimensional (3D) Matrigel cultures. These cells exhibit the differentiation potential of three germ layers and primordial germ cell, and display many features resembling mouse E6.5 epiblasts just before gastrulation [33]. Building on this work, we employed the similar 3D culture system to mimic the in vivo microenvironment in humans and successfully established the self‐renewal human formative pluripotent stem cell‐like cells (hfPSC‐LCs). Unlike mouse fPSCs, which require activation of the Nodal signalling pathway for their establishment and maintenance [33], hfPSC‐LCs exhibited little dependence on Nodal signalling during their derivation. In addition, the derivation time was another notable difference between hfPSC‐LCs and mouse fPSCs, probably reflecting the distinct developmental timeline between these two species. Similar to mouse fPSCs, hfPSC‐LCs retained multi‐lineage differentiation capacity, including the ability to generate cells of three germ layers and hPGCLCs. Notably, hfPSC‐LCs differentiated into amnion‐like precursor cells (hALPCs), transcriptionally and morphologically similar to the human bona fide amnion – a potential lacking in mouse fPSCs. Thus, this model provides a unique tool for the investigations of human early embryogenesis.

## 2 Materials and Methods

### 2.1 Conventional hESCs, hiPSCs, and Naïve hESCs Cultures

Conventional hESCs (H1, H9, H9‐GFP, and WIBR3) and hiPSCs were maintained in commercial Essential 8 medium (Gibco, Cat# A1517101) on Matrigel (Corning, Cat# 356230/354230/356231) coated dishes at 37°C and 5% CO_2_. The hESCs/hiPSCs were passaged mechanically every 5–7 days by a brief DPBS (HUANKE, Cat #HK2109.21) wash followed by dissociation using EDTA (Thermo Fisher Scientific, Cat# 15575‐038).

5iLA‐Naïve hESCs (WIBR3) were maintained in medium as described by Theunissen et al. [25]. The complete medium (500 mL) consisted of: 240 mL DMEM/F12 (Gibco, Cat# 11320), 240 mL Neurobasal (Gibco, Cat# 21103), 2.5 mL N2 supplement (Gibco, Cat# 17502048), 5 mL B27 supplement (Gibco, Cat# 17504044), 1 × 10^3^ units/mL recombinant human LIF (made in‐house), 1 mM GlutaMax (Gibco, Cat# 35050‐061), 1% nonessential amino acids (Gibco, Cat# 11140‐050), 1% penicillin–streptomycin (Gibco, Cat# 15140‐122), 1% Sodium Pyruvate (Gibco, Cat# 11360‐070), 0.1 mM 2‐Mercaptoethanol (Gibco, Cat# 21985023), 50 mg/mL BSA (Sigma‐Aldrich, Cat# A4378). The medium was further supplemented with the following small molecules and cytokines: 1 μM PD0325901 (LC Laboratories, Cat# P‐9688), 1 μM IM‐12 (Enzo Life Sciences, Cat# 1129669‐05‐1), 0.5 μM SB590885 (R&D systems, Cat# 2650/10), 1 μM WH‐4‐023 (MCE, Cat# HY‐12299), 10 μM Y‐27632 (TOCRIS, Cat# 1254), 20 ng/mL Activin A (Peprotech, Cat# 100‐18B), and 1 × 10^3^ units/mL recombinant human LIF (made in‐house). Naïve hESCs were routinely cultured on MEF in 5% O_2_ and 5% CO_2_.

PXGL‐Naïve hESCs (H9) were propagated in N2B27 with 1 mM PD0325901, 2 mM XAV939 (Sigma‐Aldrich, Cat# X3004), 2 mM Gö 6983 (MCE, Cat# HY‐13689), and 1 × 10^3^ units/mL recombinant human LIF (made in‐house) on irradiated MEF. ROCK inhibitor (Y‐27632) and Matrigel (1:100) were added to medium during replating. Cells were cultured in 5% O_2_, 5% CO_2_ in a humidified incubator at 37°C and passaged by dissociation with Accutase (Gibco, Cat# A11105‐01) every 3–5 days following the previous work [46].

### 2.2 Derivation and Maintenance of hfPSC‐LCs From Naïve hESCs, Conventional hESCs and hiPSCs

5iLA‐naïve hESCs (WIBR3), PXGL‐Naïve hESCs (H9), conventional hESCs (H1, H9, and WIBR3), and hiPSCs were treated with Accutase (Gibco, Cat# A11105‐01) or 50% TrypLE Express (Gibco, Cat# 12605010) (diluted in DPBS) for 3 min at 37°C to dissociate them into single cells. The cells were collected by centrifugation at 300 g/min for 3 min. Subsequently, the cells were re‐suspended in hfPSC‐LCs medium at a density of 2 × 10^5^ cells per millilitre. Then 100 μL of cell suspension was mixed with an equal volume of Matrigel. This cell‐Matrigel mixture was plated and incubated at 37°C for 1 h to allow Matrigel solidification. Following solidification, 3 mL of hfPSC‐LCs medium was added to the 35 mm cell culture dish. The hfPSC‐LCs medium (50 mL) consisted of: 50 mL E8 complete medium, 2 μM IWP2 (Merck, Cat# 681671), 1% HSA (Sigma, Cat# A1653), and 10 μM Rho‐associated kinase inhibitor Y27632 (TOCRIS, Cat# 1254) or 10% CloneR (STEM CELL, Cat# 05888). CloneR or HSA were removed from the medium 24 h later.

For the propagation of hfPSC‐LCs, the colonies were re‐suspended and washed once with pre‐cold DPBS. Then the cells were digested with 50% TrypLE Express for 1 min at 37°C to dissociate into single cells, and subsequently the cells were collected by spin down at 1000 g/min for 3 min. These cells were re‐suspended in hfPSC‐LCs medium and added with equal volume of Matrigel. Cells in Matrigel were plated and incubated at 37°C for 30 min. After Matrigel solidification, the cells were added with pre‐warmed hfPSC‐LCs medium and cultured at 37°C and 5% CO_2_. Generally, hfPSC‐LCs were propagated every 4–6 days. The cryopreservation medium for hfPSC‐LCs contained 90% FBS (Gibco, Cat# 14000‐044) and 10% Dimethylsulfoxide (DMSO, Sigma‐Aldrich, Cat# D8418).

### 2.3 Derivation of hfPSC‐LCs From Human Blastocysts

Human embryos were thawed as per the manufacturer's instructions and cultured for two days in a suspension manner with N2B27 medium in 5% CO_2_ and 5% O_2_ at 37°C. ICMs were isolated through mechanical dissociation and were cultured in 3D‐Matrigel conditions. After 5–8 days, the resulting outgrowths were mechanically digested into single cells using 50% TrypLE Express and re‐plated onto a new dish. Typical hfPSC‐LCs colonies emerged at this generation.

### 2.4 Cardiomyocytes Differentiation

Cardiomyocytes induction was performed as previously established protocols [33, 47]. Briefly, 3000–5000 single cells of hfPSC‐LCs were cultured in 35 mm cell cultured dishes for 3–4 days, after which IWP2 was removed from the hfPSC‐LCs medium, and the cells were maintained in E8 complete medium for an additional 2–3 days. Subsequently, the medium was changed to RPMI 1640 medium (1×) (Gibco, Cat# 11875‐093) supplemented with 4 mM VC (Vitamin C, Sigma Aldrich, Cat# A7506), B27 supplement minus insulin (Gibco, Cat# A1895601) (Basal Medium 1, BM1) and 3 μM Chir99021 (LC Laboratories, Cat# C‐6556) for the first day of differentiation. Following this, Chir99021 was withdrawn from the differentiation medium and continuous cultivation took place for another 2 days in the BM1. Next, 10 μM XAV939 was introduced into the BM1 medium and removed from it after 24 h. Upon the appearance of beating colonies, the medium was refreshed every day with RPMI 1640 supplemented with 4 mM VC and normal B27 supplement plus insulin (Gibco, Cat# 17504044) (Basal Medium 2, BM2).

Mouse cardiomyocyte induction from 2D‐adapted fPSCs was carried out according to the method we previously reported [33]. In short, single cells of fPSCs were plated into a Matrigel‐coated 6‐well plate and cultured with mouse fPSCs medium for 3–5 days, then the medium was changed to mouse cardiomyocyte induction medium.

### 2.5 Neural Induction

Neural induction of hfPSC‐LCs was optimised based on previously reported methods [48]. Firstly, the hfPSC‐LCs medium was replaced with N2B27 basal medium plus 10 nM SB431542 (MCE, Cat# HY‐10431) and 30 nM LDN193189 (MCE, Cat# HY‐12071) on day 5 and the cells were cultured in this condition for another 5 days. At this stage, SOX1 positive neural precursor cells were obtained. Subsequently, SB431542 was withdrawn from the medium, and the cells were maintained in this system with daily medium changes until typical neurons were observed (almost 7–10 days).

Neural precursor cells induction from 2D‐adapted fPSCs was carried out following the previously reported method [33, 49]. In brief, fPSCs (46C, Sox1‐GFP) were digested into single cells and seeded on a Matrigel‐coated 12‐well plate. Then they were cultured with fPSCs medium for 2–3 days, and the medium was changed to N2B27 medium supplemented with 2 μM PD0325901 (LC Laboratories, Cat# P‐9688) for the first day, followed by culturing in N2B27 medium for another one day.

### 2.6 Definitive Endoderm Differentiation

The induction of definitive endoderm cells from hfPSC‐LCs was carried out as previously described [50]. On day 3, hfPSC‐LCs were directed to definitive endoderm cells using medium containing 100 ng/mL Activin A (Peprotech, Cat# 100‐18B) and 3 μM Chir99021 (LC Laboratories, Cat# C‐6556) for the first day, followed by medium containing only 100 ng/mL Activin A for the subsequent two days. FOXA2 and SOX17 double‐positive cells emerged following this protocol.

### 2.7 hPGCLCs Induction

The induction of iMeLCs was carried out based on previously published work. The iMeLCs were induced by plating 1.0 × 10^5^ single cells of hESCs (H1 and H9) onto a Matrigel coated 12‐well cell culture plate in G‐MEM medium (1×) (Gibco, Cat# 11710‐035) supplemented with 15% KSR (Gibco, Cat# 10828028), 1 mM sodium pyruvate (Gibco, Cat# 11360070), 0.1 mM non‐essential amino acids (Gibco, Cat# 11140050), 0.1 mM 2‐Mercaptoethanol (Invitrogen, Cat# 21985‐023), 2 mM L‐glutamine (Gibco, Cat# 35050061) and 100 units/mL penicillin, 0.1 mg/mL streptomycin (Gibco, Cat# 15140122) (GK15 medium) supplemented with 50 ng/mL of Activin A (PeproTech, Cat# 120‐14), 3 μM of CHIR99021 (LC Laboratories, Cat# C‐6556), and 10 μM of the ROCK inhibitor (TOCRIS, Cat# 1254) [51].

The hPGCLCs were induced by plating 3000 single cells of iMeLCs, hfPSC‐LCs or 3–5 intact colonies of hfPSC‐LCs at day 5 into low cell attachment U‐bottom 96‐well plate (Corning, Cat# 7007) in GK15 medium, supplemented with 1000 U/mL of LIF (made in‐house), 200 ng/mL of BMP4 (R&D Systems, Cat# 315‐27), 100 ng/mL of SCF (R&D Systems, Cat# 455‐MC‐010), 50 ng/mL of EGF (R&D Systems, Cat# 2028‐EG), and 10 μM of the ROCK inhibitor (TOCRIS, Cat# 1254).

mPGCLCs induction from 2D‐adapted fPSCs was operated according to the published method [52]. Briefly, single cells of fPSCs were plated on a Matrigel‐coated 12‐well plate and cultured with fPSCs medium for 3–4 days. Subsequently, the 2D‐adapted fPSCs were digested into single cells and aggregated in a low cell attachment U‐bottom 96‐well plate.

### 2.8 Human Amnion‐Like Precursor Cells Established From hfPSC‐LCs

Firstly, hfPSC‐LCs were dissociated into single cells using 50% TrypLE Express and seeded in 3D‐Matrigel. Then they were cultured in medium (50 mL) including: 50 mL N2B27 basal medium, 1 μM PD0325901 (LC Laboratories, Cat# P‐9688) and 1 μM A83‐01 (Selleck, Cat# S7692) [38]. The culture medium was refreshed every other day, and the prepared medium could be stored in refrigerator for up to one week. The hALPCs could be passaged every 8–10 days. The detailed protocols are as follows: hALPCs colonies at day 8–10 were re‐suspended with pre‐cold DPBS and collected by centrifugation at 3000 g/min for 3 min. Then hALPCs were digested with 50% TrypLE Express and re‐plated in Matrigel for another passage.

### 2.9 Immunostaining Analysis

The cells were fixed with 4% paraformaldehyde (PFA) (Sigma‐Aldrich, Cat# 158127) for 15 min at room temperature. After washing 3–5 times with DPBS containing 0.1% Triton X‐100 (Amresco, Cat# 0694), the samples were penetrated with DPBS containing 1% Triton X‐100 for 15 min and blocked with 0.5% BSA (Sigma‐Aldrich, Cat# A4378) in DPBS at room temperature for 1 h. Then the samples were incubated with the primary antibodies overnight at 4°C. After washing three times with DPBS, the samples were incubated 1 h with the second antibodies at room temperature, protected from light. Nuclear counterstaining was achieved with Hoechst 33342 (Invitrogen, Cat# H3570), while F‐actin was visualised using Phalloidin conjugates (Alexa Fluor 594/488, Thermo Fisher Scientific, Cat# A12381/A12379, 1:200). Images were acquired with Zeiss microscope (LSM 880 or LSM 880 Fast Airyscan; Carl Zeiss). The primary antibodies included: rabbit anti‐Ezrin (Abcam, Cat# Ab76247, 1:200); rabbit anti‐SOX1 (Cell Signalling Technology, Cat# 4194S, 1:200), rabbit anti‐Tuj1 (Sigma‐Aldrich, Cat# T2200, 1:100), goat anti‐Oct4 (Santa Cruz Biotechnology; Cat# sc‐8628, 1:200), goat anti‐Nanog (R&D systems; Cat# AF1997, 1:200), mouse anti‐Sox2 (Cell Signalling Technology; Cat# 4900S, 1:200), mouse anti‐cardiac troponin T (cTnT, Thermo Fisher Scientific, Cat#MS‐295‐P0, 1:400), mouse anti‐α‐actinin (Sigma‐Aldrich, Cat# A7732, 1:500), rabbit anti‐myosin light chain (MLC) 2v (ProteinTech Group, Cat# 10906‐1‐AP, 1:50), rabbit anti‐Foxa2 (Cell Signalling Technology, Cat# 8186S, 1:200), goat anti‐Sox17 (R&D systems, Cat# AF1924, 1:200), rabbit anti‐STELLA (Santa Cruz Biotechnology, Cat# sc‐67,249, 1:200), goat anti‐Otx2 (R&D systems, Cat# AF1979, 1:100), rabbit anti‐E‐Cadherin (Santa Cruz Biotechnology, Cat# sc‐7870, 1:200), rabbit anti‐KRT18 (ABclonal, Cat# A19778), rabbit anti‐TFAP2C (Cell Signalling Technology, Cat# 2320S, 1:200), mouse anti‐TFAP2A (Santa Cruz Biotechnology, Cat# sc‐12,726, 1:200), rabbit anti‐GATA2/3 (Abcam, ab182747, 1:200), rabbit anti‐cleaved Caspase3 (Cell Signalling Technology, Cat# 9664, 1:200), rabbit anti‐T/Brachyury/TBXT (ABclonal, Cat# A5078, 1:200), rabbit anti‐Klf4 (ABclonal, Cat# A13673, 1:200), PE anti‐human SUSD2 Antibody (BioLegend, Cat# 327406, 1:200). The secondary antibodies from Jackson ImmunoReseach included: Alexa Fluor 488 AffiniPure Donkey Anti‐Rabbit IgG (H + L) (Cat# 711‐545‐152, 1:200); Alexa Fluor 594 AffiniPure Donkey Anti‐Rabbit IgG (H + L) (Cat# 711‐585‐152, 1:200); Cy5 AffiniPure Donkey Anti‐Rabbit IgG (H + L) (Cat# 711‐175‐152, 1:200); Alexa Fluor 488 AffiniPure Donkey Anti‐Mouse IgG (H + L) (Cat# 711‐545‐150, 1:200); Alexa Fluor 647 AffiniPure Donkey Anti‐Mouse IgG (H + L) (Cat# 715‐605‐151, 1:200); Alexa Fluor 594 AffiniPure Donkey Anti‐Mouse IgG (H + L) (Cat# 715‐585‐150, 1:200); Alexa Fluor 488 AffiniPure Donkey Anti‐Goat IgG (H + L) (Cat# 705‐545‐003, 1:200).

### 2.10 Flow Cytometry Analysis

For the analysis of hPGCLCs induction from iMeLCs and hfPSC‐LCs, hPGCLCs aggregations were dissociated into single cells by 0.05% Trypsin–EDTA for 7–8 min at 37°C, with gentle pipetting every 3 min during this process. The cells were then stained with APC‐conjugated anti‐CD326 (BioLegend, Cat#324207, 1:100, EpCAM) and BV421‐conjugated anti‐CD49f (BioLegend, Cat#313623, 1:100, INTEGRIN 6) antibodies on ice for 15 min. After washing with GK15 basic medium, the cell suspension was filtered with the cell strainer cap (Falcon Cell Strainers, Cat# 352235) and immediately analysed by flow cytometry (BECKMAN COULTER Immunofluorescence analysis Life Sciences). The un‐dyed single cells of hPGCLCs induction from hfPSC‐LCs served as a negative control. Data analysis was performed with FlowJo_ V10 software (Beckman Coulter MoFlo XDP).

For the quantitative analysis of Sox1^+^ neural precursor cells induction from 2D‐adapted 46C‐fPSCs (Sox1‐GFP‐fPSCs), after two days induction, cells were digested into single cells with 0.25% Trypsin–EDTA for 7–8 min at 37°C and immediately analysed the proportion of GFP‐positive cells by flow cytometry. The undifferentiated 46C‐fPSCs served as a negative control.

### 2.11 Time‐Lapse Imaging

Time‐lapse imaging of the beating cardiomyocyte colonies was performed using an imaging system. The images were captured over a 3‐min period. The acquired data were subsequently processed with Imaris Viewer software.

### 2.12 Intracellular Ca2^+^ Measurements

For intracellular Ca2^+^ measurements, the contracting cardiomyocytes were labelled with Fluo‐3 AM (Beyotime, Cat# S1056, 1:5000) in fresh cardiomyocytes differentiation medium at 37°C and 5% CO_2_ for 1 h. The cells were washed twice with fresh medium and the Fluo‐3 AM fluorescence of Ca2^+^ in the cells was recorded by ultra‐high resolution live cell confocal microscope (Andor company, Dragonfly 200) system.

### 2.13 Quantitative RT‐PCR

The mRNA was isolated from the cells and embryonic fragments of epiblast with RNAzol (Mrcgene, Cat# RN190) following the manufacturer's instructions. The concentration of mRNA was measured by NanoDrop 2000 (Thermo Fisher Scientific). For each sample, 500 ng mRNA was transcribed into cDNA with PrimeScript RT Reagent Kit (TaKaRa, Cat# RR037A). Quantitative PCR (qPCR) was performed with EvaGreen 2× qPCR MasterMix (ABM, MasterMix‐S) on LightCycler 480 II (Roche). All results were repeated with at least three independent samples. β‐actin was used as an internal normalisation control. The 2^−ΔCt^ method was used for data analysis [53]. All primer sequences for qPCR were listed in Table S1.

### 2.14 Calculation of the Cell Doubling Time

The doubling time of hfPSC‐LCs and hESCs was calculated using the doubling time online calculator (https://www.doubling‐time.com/compute.php?lang=en). Briefly, 4000 single cells of hESCs (H1) were seeded on Matrigel coated 12‐well plate and cultured for 4 days, while 5000 single cells of hfPSC‐LCs (H1 and H9) were cultured in 3D‐Matrigel for 5 days. Subsequently, the cells were collected and their doubling time was calculated. All experiments were performed with three independent biological replicates.

### 2.15 Cloning Efficiency Analysis of hESCs and hfPSC‐LCs

For the analysis of cloning efficiency of hESCs, 4000 single cells of hESCs (H1) were seeded on Matrigel coated 12‐well cell culture plate in triplicates. The cells were cultured for 4 days and then counted the number of colonies. For the analysis of cloning efficiency of hfPSC‐LCs, 3000 single cells of hfPSC‐LCs were plated on 35 mm cell culture dishes in triplicates. The cells were maintained in E8 + IWP2 + Y27632, E8 + IWP2 + 1% HSA, E8 + IWP2 + 1% HSA + Y27632, and E8 + IWP2 + 10% CloneR medium for 5 days, after which colonies were enumerated to determine cloning efficiency.

### 2.16 Alkaline Phosphatase Staining

Alkaline phosphatase staining was performed with BCIP/NBT Alkaline Phosphatase Colour Development Kit (Beyotime, Cat# C3206) following the manufacturer's instructions. Briefly, the hfPSC‐LCs colonies at day 5 were fixed with 4% PFA for 30–60 s at room temperature. Following three times washes with DPBS, the cells were incubated with BCIP/NBT solution for 30 min at room temperature in the dark. Ultimately, the images were captured with a CCD camera.

### 2.17 Karyotype Analysis

The hfPSC‐LCs at day 4–5 were synchronised at metaphase through treatment with 0.02 mg/mL colchicine (Sigma‐Aldrich, Cat# C9754) at 37°C and 5% CO_2_ for 1 h. The metaphase cells were dissociated into single cells and incubated with hypotonic KCl solution (0.56%) for 6 min. Then, the cells were fixed four times with 5 mL ice‐cold fixative solution (methanol: acetic acid = 3:1) and collected. Cell pellets were re‐suspended with 200 μL fixative solution, spread onto the ice‐cold slides. Subsequently, slides were counterstained with 5% Giemsa solution (Sigma‐Aldrich, Cat #GS500) for 120 min at room temperature for G‐banding visualisation. Ultimately, the karyotype was detected by mercury lamp and counted by Videotest software.

### 2.18 The Establishment of *SNAI1* ‐mCherry Reporter Cell Line

A *SNAI1*–mCherry knock‐in (KI) reporter hESCs was generated by CRISPR/Cas9‐mediated genome editing to knock‐in a P2A‐mCherry‐PGK‐Neo reporter cassette before the STOP codon of the endogenous *SNAI1* locus. Briefly, the guide RNA (gRNA) was PCR amplified (Forward: 5′‐CACCGgatgtccccgctgaccctcg‐3′; Reverse: 5′‐AAACcgagggtcagcggggacatcC‐3′) and cloned into PX459 vector. The pUC19‐*SNAI1*‐P2A‐mCherry‐PGK‐Neo donor template plasmid was constructed by PCR‐based cloning. The donor template contained a 5′ homology arm of 814 bp (Forward: 5′‐gccccaggagtggcctaacc‐3′; Reverse: 5′‐gcggggacatcctgagcagc‐3′) and a 3′ homology arm of 890 bp (Forward: 5′‐ccctcgaggctccctcttcc‐3′; Reverse: 5′‐gtcccctcctcccttaccaa‐3′). Per 1 × 10^5^ hfPSC‐LCs were electroporated with 0.45 μg of the *SNAI1*–end PX459 plasmid, 0.15 μg of the pUC19‐*SNAI1*‐P2A‐mCherry‐PGK‐Neo donor template plasmid. After the cells were selected by G418 (400 μg/mL) for 5 days, colonies were picked and genotyping with the primers of forward: 5′‐ACAGACAAGGAGATTTGGGATTAC‐3′; reverse: 5′‐AGGGGCCACCAAAGAACGGAGC‐3′.

### 2.19 Annexin V‐FITC Apoptosis Detection Kit Staining

The Annexin V‐FITC Apoptosis Detection Kit (Beyotime, Cat# C1062S) is used to detect phosphatidylserine (PS) externalisation on the cell membrane during apoptosis using FITC‐labelled recombinant human Annexin V. Annexin V selectively binds to PS, a phospholipid normally confined to the inner leaflet of the plasma membrane. During early apoptosis, PS is rapidly translocated to the outer membrane surface across various cell types. By utilising Annexin V conjugated to the green‐fluorescent FITC probe, PS externalisation—a key marker of early apoptosis—can be directly observed via fluorescence microscopy. Furthermore, co‐staining with propidium iodide (PI) allows for the differentiation of necrotic cells.

### 2.20 RNA Sequencing and Data Analysis

Total RNA was isolated from conventional hESCs, hfPSC‐LCs, hESCs cultured in suspension manner, 2D‐adapted fPSCs, and hALPCs using TRIzol Reagent (MRC, Cat# RN 190). Genomic DNA (gDNA) contamination was eliminated from the total RNA by the digestion of RQ1 DNase (Promega, Cat# M610A) for 1 h at 37°C. RNA integrity was verified by Bioanalyzer 2100 (Agilent Technologies) with RNA Integrity Number (RIN) > 8.0. For library preparation, 3 μg total RNA was used to construct the RNA sequencing libraries. RNA‐seq libraries were sequenced on Illumina NovaSeq X.

Low‐quality RNA sequencing reads and adapters were removed by cutadapt v1.11 and then the clean reads were aligned to the human cDNA database (hg19) using HISAT2 (v2.2.1) [54]. The gene counts were calculated with gene annotation from the UCSC database using FeatureCounts (v2.0.1) ([55]). TPM (transcripts per million) was introduced to calculate gene expression value. DEGs were identified by the R DESeq package (v1.24.0) [56], and the threshold of *p*‐value was 0.05. Heatmaps of differentially expressed genes were further clustered by hierarchical clustering and then were visualised by the R pheatmap package (v1.0.12). PCA analysis was performed and visualised by using all expressed genes (http://www.r‐project.org). DAVID online analysis (DAVID Bioinformatics Resources 6.8) was used for GO enrichment analysis.

### 2.21 ATAC‐Seq Data Processing

After removal of reads with poor quality and adapters, reads from ATAC‐seq datasets were mapped to the human genome (hg19) using Bowtie2 v. 2.2.2 [57]. The UCSC genome browser was used to visualise ATAC‐seq data.

### 2.22 MethylC‐Seq Library Generation and Data Processing

Genomic DNA (gDNA) was isolated using phenol/chloroform extraction and subsequently ethanol precipitation. Whole‐Genome Bisulfite Sequencing (WGBS) was conducted as described previously. Paired‐end DNA sequencing (2 × 150 nucleotides) at the Illumina NovaSeq X platform was used. After trimming low quality sequencing reads and adapters, the clean reads were mapped to hg19 by Bismark (v0.23.1) [58] following the default parameters. Unconverted reads, multiple mapped reads, and duplicate reads introduced by PCR were also removed.

### 2.23 Single Cell Analysis

Single cells were obtained from hfPSC‐LCs colonies after digestion by 50% TrypLE Express at 37°C for 1 min. And then the transcriptome library construction was performed according to the protocol described previously [59]. In total, 8798 cells were estimated and 4066 genes per cell were detected.

## 3 Results

### 3.1 The Establishment of Human Formative Pluripotent Stem Cell‐Like Cells (hfPSC‐LCs)

To determine whether the formative pluripotency in mice could be extrapolated to humans, we first seeded hESCs into 3D‐Matrigel and cultured them in mouse fPSCs medium [33]. However, directly applying the mouse fPSCs culture system to human cells was not an effective strategy, as single cells of hESCs failed to produce colonies resembling those of mouse fPSCs. Given hESCs maintained in Essential 8 (E8) medium are generally considered to represent an intermediate pluripotent state [60], we employed E8 medium as the basal medium to establish human formative pluripotent cells from conventional H1 and H9 hESCs. However, E8 medium alone was insufficient for maintaining proper colony morphology and failed to suppress the expression of mesodermal marker TBXT (Figure S1A,B), suggesting that additional factors are required to establish human formative pluripotent cells. Further investigation demonstrated that pharmacological inhibition of the Wnt/β‐catenin pathway using IWP2, a Wnt signalling inhibitor, more effectively maintained clonal morphology for both H1 and H9 cell lines compared to XAV939 (which stabilises axin2 to promote β‐catenin degradation) (Figure S1A–D). When N2B27 was used as the basal culture medium, this system failed to support the formation of rosette structures (Figure S1E,F), and resulted in widespread apoptosis or necrosis upon passaging (Figure S1G). The morphological difference between the two culture systems was easily observed at low‐magnification images (Figure S1H,I).

Notably, a synergistic survival cocktail combining human/bovine serum albumin (HSA/BSA, 1%) with Y‐27632 (10 μM) significantly enhanced single‐cell viability of hfPSC‐LCs in 3D‐Matrigel culture, achieving survival rates comparable to commercial reagents (CloneR) (Figure S2A,B). However, Rho/ROCK inhibitor alone [61] could not reverse the outcome of cell death in 3D‐Matrigel culture condition for the single‐cell viability of these cells (Figure S2A,B). Under the optimised 3D Matrigel conditions, the mixed single‐cell suspensions of GFP^+^‐ and GFP^−^‐hESCs gave rise to GFP^+^‐ and GFP^−^‐rosette colonies after 3 days in culture (Figure S2C), confirming the single‐cell formation of these colonies. Therefore, the combination of E8 basal medium and the small molecule inhibitor IWP2 enabled the establishment of stably propagated rosette‐like colonies from single cells of hESCs. Based on analogy with mouse fPSCs [33], these self‐renewing, rosette‐like colonies were accordingly designated as human formative pluripotent stem cell‐like cells (hfPSC‐LCs) (Figure S2D).

**FIGURE 1:**
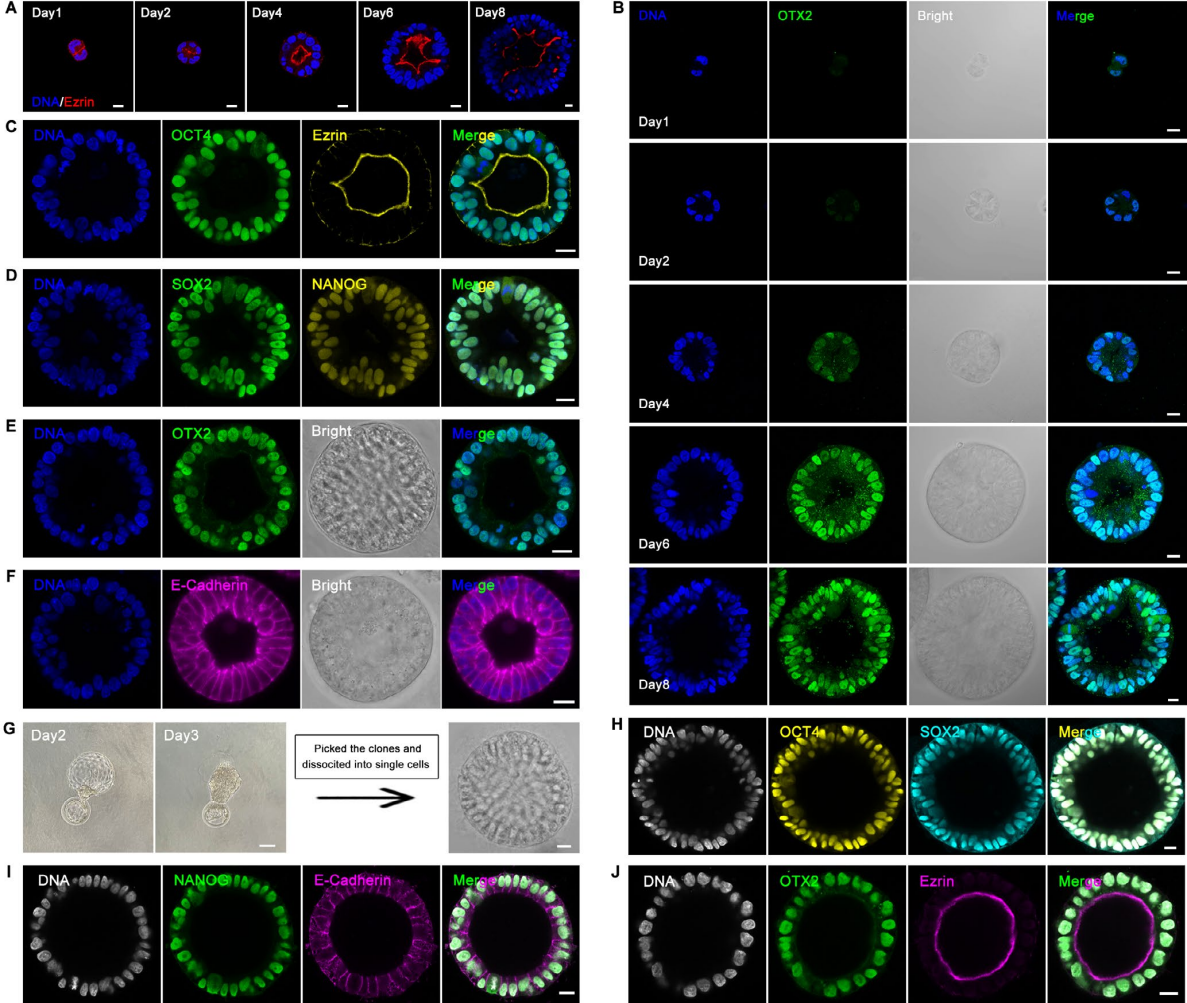
The establishment of hfPSC‐LCs from naïve hESCs and human blastocyst. (A) The transition process of hfPSC‐LCs from naïve hESCs (5iLA, WIBR3) was recorded by the immunostaining of apical lumen (Ezrin, red) at days 1, 2, 4, 6, and 8. DNA was stained with Hoechst 33342 (blue). Scale bars, 20 μm. (B) Immunofluorescence staining was performed using anti‐OTX2 antibody (green) to monitor OTX2 protein level during naïve hESCs to hfPSC‐LCs (WIBR3) conversion at designated time points (Day 1/Day 2/Day 4/Day 6/Day 8). DNA was stained with Hoechst 33342 (blue). Scale bars, 20 μm. (C–F) Immunostaining of the expression of OCT4/POU5F1 (green), SOX2 (green), NANOG (yellow), OTX2 (green), Ezrin (yellow), and E‐Cadherin (magenta) in the long term propagated hfPSC‐LCs (WIBR3). DNA was stained with Hoechst 33342 (blue). Scale bars, 20 μm. (G) The frozen human embryos were thawed using G‐MOPS PLUS handling medium and then cultured in suspension manner with N2B27 medium for two days. The bright‐field (BF) image showing human blastocyst cultured in 3D‐Matrigel supplemented with hfPSC‐LCs medium for another two or three days as well as the eventually established hfPSC‐LCs line. Scale bars, 20 and 50 μm, respectively. (H–J) The immunostaining of the expression of OCT4/POU5F1 (yellow), SOX2 (cyan), NANOG (green), E‐Cadherin (magenta), OTX2 (green), and Ezrin (magenta) in hfPSC‐LCs established from human blastocyst. DNA was stained with Hoechst 33342 (blue). Scale bars, 20 μm.

To monitor the transition from ground to formative state in humans, naïve hESCs [25] were adopted to produce hfPSC‐LCs using this system. Immunostaining results showed that naïve hESCs cultured in the 5iLA system expressed human naïve pluripotency factors (KLF4 and SUSD2) and canonical pluripotent related markers (SOX2, OCT4/POU5F1, and NANOG), while lacking formative pluripotent regulator OTX2 expression (Figure S3A–D). Time‐course recording documented the dynamic morphological transformation of naïve hESCs (5iLA) into hfPSC‐LCs over 8 days, as shown by staining for DNA and polarity regulator Ezrin (Figures 1A and S4A). During early polarisation phases (two‐cell stage), the apical accumulation of Ezrin was observed at the cell–cell interfaces. Lumen formation was initiated around day 3, with subsequent cavity expansion preceding complete morphological conversion of epithelial‐like cells. Notably, the formative marker OTX2 was expressed during the transition from naïve to formative state (Figure 1B). The common pluripotent factors of OCT4/POU5F1, SOX2, and NANOG, and the formative marker OTX2 were consistently expressed in the propagated hfPSC‐LCs (Figure 1C–E). Meanwhile, adherens junction related protein E‐Cadherin/CDH1 was concentrated on the cell membrane of hfPSC‐LCs (Figure 1F). Similarly, we also established hfPSC‐LCs from PXGL‐naive hESCs (Figure S4B) [46], and the hfPSC‐LCs obtained from PXGL‐hESCs also expressed common pluripotent markers (OCT4/POU5F1 and NANOG) as well as formative marker OTX2 (Figure S4C–E). In addition, hfPSC‐LCs could also be established from hiPSCs. These cells exhibited Ezrin and E‐Cadherin localization patterns and expressed pluripotency markers (OCT4/POU5F1, SOX2, NANOG, and OTX2) at levels similar to those in hfPSC‐LCs derived from naïve hESCs (Figure S5A–C).

Notably, we also established one hfPSC‐LCs line from three human blastocysts (1/3) through a stepwise protocol. Following thawing, human blastocysts underwent 48‐h suspension culture in N2B27 medium before being transferred to Matrigel and cultured in the maintenance medium of hfPSC‐LCs. Embryos hatching from zona pellucida were completed within 72 h post‐plating, and subsequent outgrowths were mechanically dissociated into single cells between day 5 and 8 for clonal expansion (Figure 1G). The blastocyst‐derived hfPSC‐LCs exhibited morphological features indistinguishable from those of human PSCs‐derived counterparts (Figure 1G). The immunostaining confirmed that OCT4, SOX2, NANOG, E‐CADHERIN, as well as OTX2 were also expressed in human blastocyst‐derived hfPSC‐LCs (Figure 1H–J).

As pluripotent stem cells, hfPSC‐LCs colonies exhibited alkaline phosphatase activity (Figure S6A) and exhibited a proliferation rate comparable to conventional hESCs maintained in E8 medium, with a doubling time of 25–30 h (Figure S6B). Moreover, hfPSC‐LCs could be resuscitated efficiently after multiple freeze–thaw cycles and maintained normal karyotype through at least 36 passages (Figure S6C). Similar to hESCs at passage 55 (*n* = 12), hfPSC‐LCs derived from hESCs at passage 33 maintained a stable karyotype after an additional 23 (*n* = 12) and 36 (*n* = 11) passages. Moreover, hfPSC‐LCs generated from hiPSCs also exhibited a normal karyotype after 32 passages (*n* = 20) (Figure S6C). Collectively, using this optimised protocol, we successfully generated hfPSC‐LCs from naïve hESCs, conventional hESCs, hiPSCs, and human blastocysts, achieving robust single‐cell propagation for over 40 passages.

### 3.2 hfPSC‐LCs Harbour Unique Characteristics of Transcriptome and Epigenome

**FIGURE 2:**
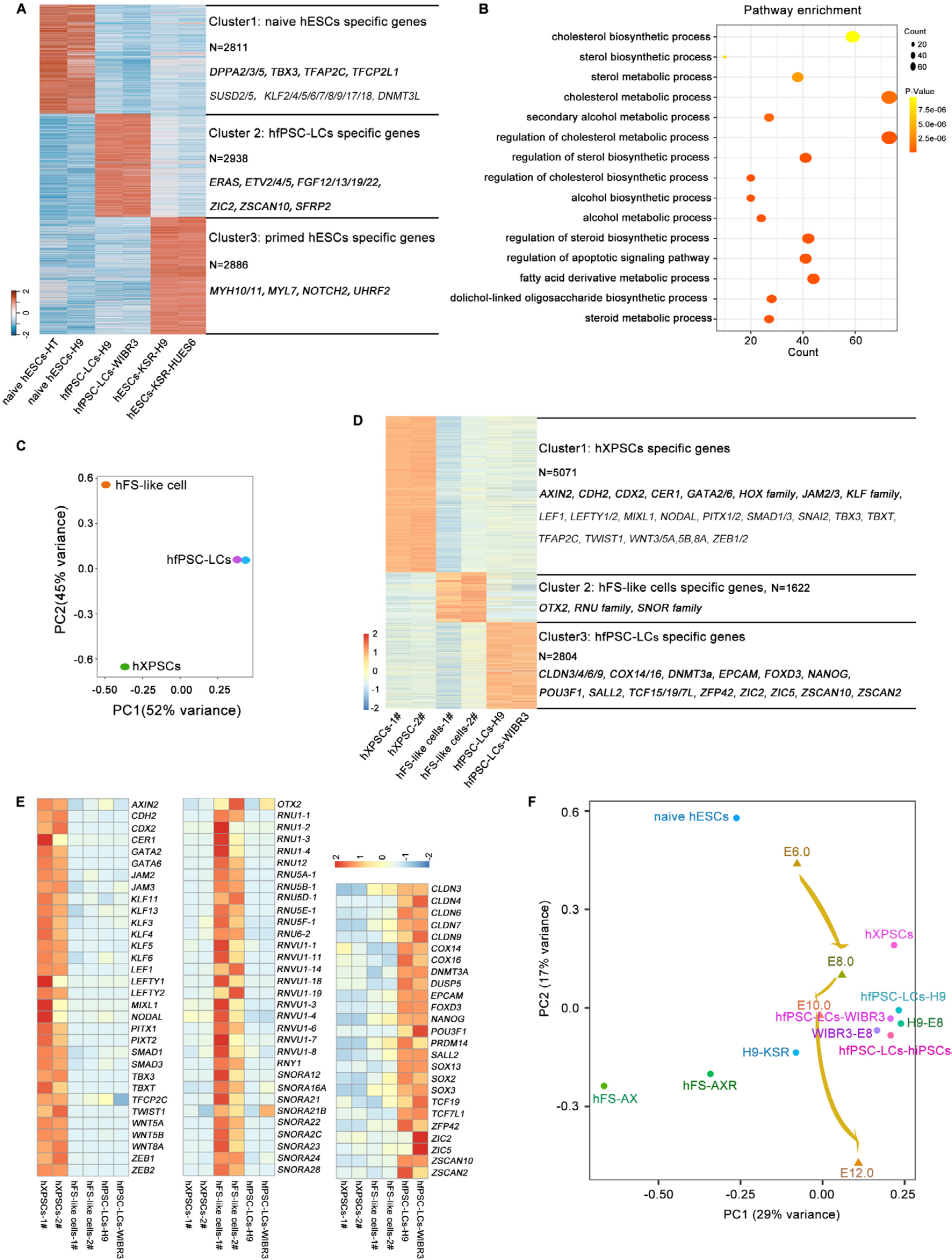
hfPSC‐LCs harbour unique formative‐like pluripotency features. (A) Heatmap of the highly expressed genes (N, number of genes) in naïve hESCs [62], hfPSC‐LCs, and primed hESCs [60] (fold change ≥ 1.5). Representative genes of different clusters were selected and listed on the right. (B) GO enrichment term results of the highly expressed genes in hfPSC‐LCs. (C) The PCA plot of RNA‐seq data of hfPSC‐LCs (H9 derived hfPSC‐LCs at Passage 6 and WIBR3 derived hfPSC‐LCs at Passage 6) and the published RNA‐seq data for hFS‐like cells [29] as well as hXPSCs [34]. (D) Heatmap of the highly expressed genes (N, number of genes) in hXPSCs, hFS‐like cells, and hfPSC‐LCs (fold change ≥ 1.5). Representative genes were listed on the right. (E) Heatmap showing the relative expression of representative genes expressed in hXPSCs, hFS‐like cells as well as hfPSC‐LCs. (F) PCA of RNA‐seq data of epiblast cells of E6.0/E8.0/E10.0/E12.0 human embryos [11] in combination with naïve hESCs [62], H9‐KSR [60], hFS‐like‐AX/AXR [29], H9/WIBR3‐E8 medium, as well as hfPSC‐LCs‐H9/WIBR3/hiPSCs measured in this study.

To investigate the specific genes that distinguish hfPSC‐LCs from naïve and primed hESCs, we performed differential expression analysis for these cells and identified 2811 genes, 2938 genes, and 2886 genes that were highly expressed in naïve hESCs, hfPSC‐LCs as well as primed hESCs (fold change ≥ 1.5; *p*‐value < 0.05), respectively (Figure 2A, Table S2). The genes related to formative pluripotency were up‐regulated in hfPSC‐LCs, including the transcription factors *ERAS*, *ETV2/4/5*, and *ZIC2*, similar to mouse fPSCs [33]. In comparison, genes up‐regulated in naïve hESCs were naïve pluripotency related factors, including *DPPA2/3/5*, *TBX3*, *TFAP2C*, *TFCP2L1*, *SUSD2/5* as well as *KLF* family. On the other hand, the genes up‐regulated in primed hESCs included numerous signatures for lineage specification, such as *MYH10/11* and *MYL7* (Figure 2A). Gene Ontology (GO) term enrichment analysis highlighted the cholesterol and sterol biosynthetic process in hfPSC‐LCs (Figure 2B), in accordance with the mouse fPSCs [33].

We then compared the transcriptional datasets of hfPSC‐LCs with hXPSCs and hFS‐like cells [29, 34]. Principal component analysis (PCA) grouped hfPSC‐LCs apart from the other two cell types (Figure 2C). Differential expression analysis (fold change ≥ 1.5; *p*‐value < 0.05) identified 5071 genes, 1622 genes and 2804 genes up‐regulated in hXPSCs, hFS‐like cells, and hfPSC‐LCs, respectively (Figure 2D, Table S3). The hfPSC‐LCs expressed many markers, distinct from both hXPSCs and hFS‐like cells (Figure 2E). In summary, transcriptomic profiling revealed that hfPSC‐LCs possess unique molecular signatures distinguishing them from naïve hESCs, conventional hESCs, primed hESCs, and the recently characterised hXPSCs and hFS‐like cells, indicating they represent a novel formative like pluripotent state.

To systematically characterise the transcriptional landscape of hfPSC‐LCs, we further analysed the RNA‐seq datasets of hfPSC‐LCs through the PCA with previously published databases from in vivo epiblast cells (E6.0, E8.0, E10.0 and E12.0) [11] and in vitro pluripotent cells (naïve hESCs, primed hESCs, hXPSCs, and hFS‐like cells) [29, 34, 62, 63]. PCA analysis showed that the hfPSC‐LCs established from different backgrounds (WIBR3, H9, and hiPSCs) resembled epiblast cells of E10.0, while hFS‐like cells and hESCs‐KSR resembled late stage epiblast cells, and hXPSCs aligned with the epiblast cells at earlier developmental stage (pre‐E8.0) (Figure 2F). Given the similarity in culture medium between hESCs and hfPSC‐LCs, we further analysed the two cell types. Morphologically, 2D‐cultured hESCs maintained in E8 medium frequently undergo spontaneous differentiation, characterised by scattered differentiated cells surrounding the undifferentiated colonies (Figure S7A). While these spontaneously differentiated cells can be eliminated by routine passaging, the recurrent emergence of differentiated cells increases the heterogeneity of hESCs populations [64]. Comparative RNA‐seq analysis revealed distinct transcriptional profiles: the genes up‐regulated in hESCs were significantly enriched in multiple developmental processes and positive regulation of cell migration (Figure S7B), while the genes enriched in hfPSC‐LCs were predominantly associated with stem cell population maintenance and negative regulation of cell migration (Figure S7C).

**FIGURE 3:**
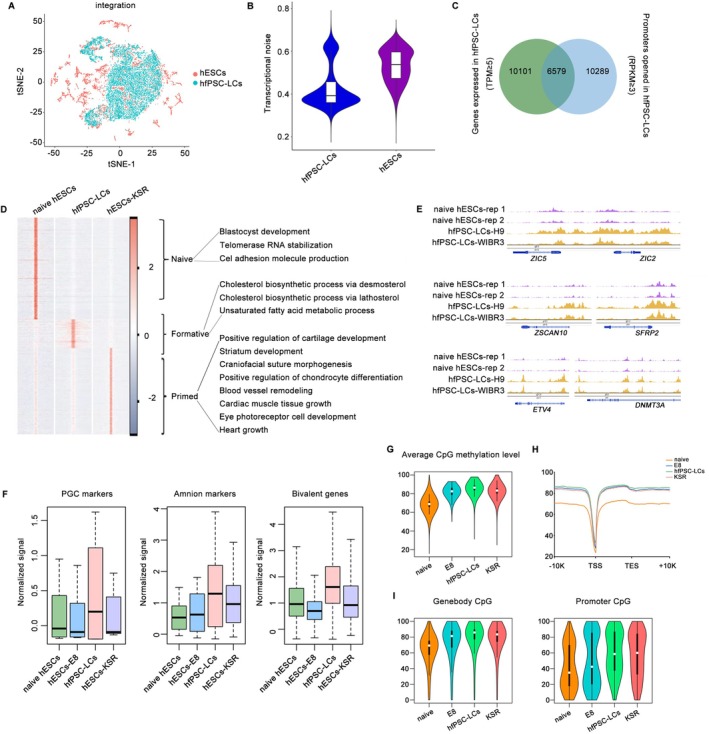
Multi‐omics Epigenome Analysis of hfPSC‐LCs. (A) The t‐SNE analysis of hfPSC‐LCs (H9 derived hfPSC‐LCs, Passage 8) single cells with published data of hESCs. hESCs and hfPSC‐LCs were represented by different colours. The single cell sequencing data for hESCs are adopted from previously published datasets [65]. (B) Violin plots showing transcriptional noise (designated as cell‐to‐cell transcriptional variability for the most variable genes) between hESCs [65] and hfPSC‐LCs. (C) Venn diagram showing the overlapping of genes expressed in hfPSC‐LCs (TPM ≥ 5) and promoters opened in hfPSC‐LCs (RPKM ≥ 3). (D) Heatmaps of ATAC‐seq peaks for genes that highly expressed in naïve hESCs [62], hfPSC‐LCs (H9 derived hfPSC‐LCs at Passage 8, WIBR3 derived hfPSC‐LCs at Passage 12), and primed hESCs [63], respectively. GO enrichment terms of the highly expressed genes in naïve hESCs, hfPSC‐LCs and primed hESCs were shown on the right. (E) The snapshots of UCSC genome browser showing the distributions of chromatin accessibility near formative pluripotent markers, *ZIC5*, *ZIC2*, *ETV4*, *SFRP2*, *ZSCAN10* and *DNMT3A*, in naïve hESCs [62] and hfPSC‐LCs. The genome browser view scales were on the basis of the global data range. (F) The average chromatin accessibility signal in the regions of PGC specific genes, amnion marker genes, and bivalent genes in naïve hESCs [62], conventional hESCs [66], hfPSC‐LCs, and primed hESCs [63]. (G) The average level of CpG methylation of naïve hESCs, conventional hESCs, hfPSC‐LCs (hfPSC‐LCs derived from H9 at P14 and hfPSC‐LCs derived from WIBR3 at P10) as well as primed hESCs. The WGBS profile of naïve hESCs, conventional hESCs, hfPSC‐LCs and primed hESCs was obtained from previous studies [60, 62]. (H) The average level of DNA methylation at TSS adjacent area (±10 kb) among naïve hESCs, conventional hESCs, hfPSC‐LCs and primed hESCs. TSS, transcription start site; TES, transcription end site. The DNA methylation data for naïve hESCs, hESCs‐E8 as well as primed hESCs were obtained from previously published datasets [60, 62]. (I) The DNA methylation at gene body and gene promoters, the DNA methylation data for naïve hESCs, hESCs‐E8 as well as primed hESCs were obtained from previously published datasets [60, 62].

To evaluate cellular homogeneity at single‐cell resolution, we performed 10 × Genomics‐based single cell RNA sequencing (scRNA‐seq) for the hfPSC‐LCs (derived from H9, Passage 8). Following standard quality control (retaining cells with ≥ 500 expressed genes), 8798 cells with a median of 4066 genes detected per cell were analysed. Compared to published hESCs datasets [65], hfPSC‐LCs formed more compact clusters in t‐SNE visualisation (Figure 3A). Transcriptional noise analysis [67] showed lower cell‐to‐cell distance variance in hfPSC‐LCs versus hESCs (Figure 3B), indicating the enhanced population uniformity of hfPSC‐LCs.

To elucidate the chromatin accessibility landscape underlying formative pluripotency and the correlation between open chromatin regions and transcriptional activity, we performed Assay for Transposase‐Accessible Chromatin sequencing (ATAC‐seq) for the hfPSC‐LCs (H9 derived hfPSC‐LCs at Passage 8 and WIBR3 derived hfPSC‐LCs at Passage 12). Global chromatin accessibility profiling revealed 6579 genes exhibiting both active transcription (TPM ≥ 5) and accessible promoters (RPKM ≥ 3) in the hfPSC‐LCs (Figure 3C, Table S4). The genes with opened chromatin in naïve hESCs [62], hfPSC‐LCs as well as primed hESCs [63] were relevant to blastocyst development, cholesterol/unsaturated fatty acid biosynthesis and lineage specification, respectively (Figure 3D). Notably, ATAC‐seq signals are enriched at the promoters of formative markers (*ZIC2/5*, *ETV4*, *SFRP2*, *ZSCAN10*, and *DNMT3A*) in hfPSC‐LCs but not naïve hESCs (Figure 3E), consistent with their high expression of these formative genes in hfPSC‐LCs (Figure 2A). Crucially, ATAC‐seq signals are constitutively enriched on genes related to PGC formation (*BLIMP1*, *TFAP2C*, *NANOS3*, *DPPA3*, *DAZL*, *DDX4*, *NANOG*, *SOX17*, and *STELLA*), amnion specification (*GABRP*, *TPM1*, *TFAP2A*, *TFAP2B*, *GATA2*, *GATA3*, *DLX5*, *HAND1*, *VTCN1*, and *ISL1*), and bivalent genes (Table S5) (Figure 3F) in hfPSC‐LCs compared with naïve hESCs, conventional hESCs, and primed hESCs [62, 63, 66]. The unique chromatin signatures of hfPSC‐LCs imply that these cells might have a superiority differentiation capacity into primordial germ cells (PGCs) and amniotic cells.

To examine the DNA methylation level of hfPSC‐LCs, we also employed the whole‐genome bisulfite sequencing (WGBS) for hfPSC‐LCs (H9 derived hfPSC‐LCs at P14 and WIBR3 derived hfPSC‐LCs at P10) and compared these data with the published datasets of naïve hESCs, conventional hESCs, and primed hESCs [60, 62]. Globally, hfPSC‐LCs exhibited high DNA methylation levels in both promoter regions and gene bodies, comparable to conventional and primed hESCs (Figure 3G–I). Collectively, these findings showed hfPSC‐LCs acquired the epigenetic signatures, which may mechanistically stabilise their formative pluripotency through coordinated chromatin remodelling.

### 3.3 Somatic and Germline Lineage Induction From hfPSC‐LCs

**FIGURE 4:**
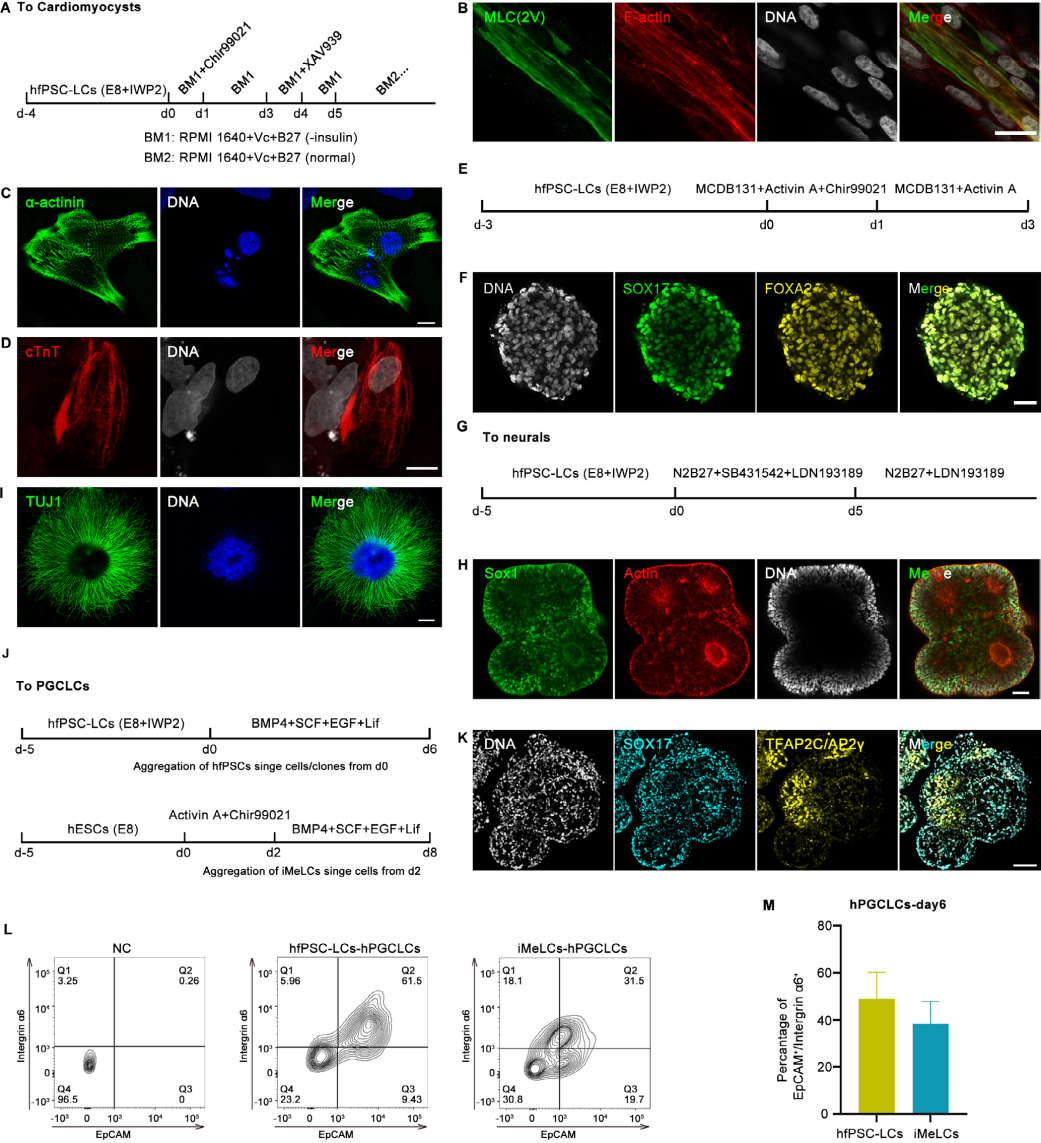
Three germ layers and primordial germ cells induction from hfPSC‐LCs. (A) The protocol used for the induction of beating cardiomyocyte‐like cells from hfPSC‐LCs. (B‐D) Cardiomyocyte‐like cells induced from hfPSC‐LCs (H9 derived hfPSC‐LCs, Passage 10) were stained with antibody for MLC (2 V) (green), α‐actinin (green), and cTnT (red). Nucleus was revealed by Hoechst 33342 staining. Scale bars, 10 μm, 20 μm, and 20 μm, respectively. (E) A schematic diagram illustrating the formation of the definitive endoderm (DE) from hfPSC‐LCs. (F) The immunofluorescent analysis of definitive endoderm cells induction from hfPSC‐LCs (H9 derived hfPSC‐LCs, P17) with antibody of SOX17 (green) and FOXA2 (yellow). DNA was stained by Hoechst 33342 staining. Scale bar, 40 μm. (G) A schematic diagram showing the differentiation of hfPSC‐LCs into neural progenitor cells (day 5) and neurons (day 10). (H) The immunofluorescent analysis of neural precursors induction from hfPSC‐LCs (H9, Passage 16) with the antibody SOX1 (green). The neural rosette was labelled by Phalloidin staining (F‐Actin, red). DNA was stained by Hoechst 33342 staining. Scale bar, 50 μm. (I) Neuron cells induction from hfPSC‐LCs (H9 derived hfPSC‐LCs, Passage 22) for ten days were labelled by TUJ1 (green). DNA was stained by Hoechst 33342 staining. Scale bar, 500 μm. (J) The model of directing generation of hPGCLCs from hfPSC‐LCs or mediating by iMeLCs. (K) The immunostaining of SOX17 (cyan) and TFAP2C/AP2γ (yellow) in hPGCLCs aggregates induction from intact hfPSC‐LCs colonies (H1 derived hfPSC‐LCs, Passage 9). DNA was stained by Hoechst 33342 staining. Scale bar, 50 μm. (L and M) FACS sorting by EpCAM and Integrin‐α6 of day 6 aggregates for the hPGCLCs induced from hfPSC‐LCs and iMeLCs. The proportion of EpCAM/Integrin‐α6 double positive cells were displayed in Figure 4M. All experiments were repeated at least three times. Error bars represented SEM.

To evaluate the differentiation of hfPSC‐LCs, we directed their commitment toward all three germ layers. For mesoderm differentiation, hfPSC‐LCs at day 4–5 were exposed to a previously optimised cardiac induction medium of mouse fPSCs (Figure 4A) [33, 47, 68]. The beating colonies emerged within 7–10 days with the proportion of approximately 53% (Figure S8A), exhibiting characteristic myofilament structure labelled by cardiomyocyte markers MLC (2 V), α‐actinin, and cTnT (Figure 4B–D) [69]. Time‐lapse microscopy captured the rhythmic contractions of these cardiomyocyte‐like colonies and recorded the typical calcium transient signals in hfPSC‐LCs‐derived cardiomyocytes (Movies S1 and S2, Figure S8B). Besides, endodermal precursors (SOX17^+^/FOXA2^+^) were generated from hfPSC‐LCs through 3‐day stimulation of Wnt/β‐catenin and Nodal/Activin A signalling pathway at an efficiency of more than 80% (Figures 4E,F, S8C) [50]. Relative to the undifferentiated hfPSC‐LCs, definitive endoderm cells induced from hfPSC‐LCs exhibited a marked upregulation of *FOXA2* and *SOX17* expression (Figures 4F, S8D,E). To examine whether hfPSC‐LCs could be used for the investigations of human gastrulation, we established a mCherry reporter line of *SNAI1*, a master regulator of epithelial‐to‐mesenchymal transition (EMT) [70]. *SNAI1‐*mCherry hfPSC‐LCs were differentiated in Advanced DMEM/F12 medium for 48 h; mCherry signal was observed to spread from the rosette‐like colonies, suggesting the migration of these cells (Figure S8F), similar to mouse fPSCs when they were released from the conditions of EMT inhibition [33]. Immunostaining for the key mesendodermal transcription factors TBXT and FOXA2 revealed that hfPSC‐LCs adopted a mesendodermal fate when they differentiated in Advanced DMEM/F12 medium for 48 h (Figure S8G). These observations suggest that hfPSC‐LCs could be used to partially mimic human gastrulation. Additionally, using the neural differentiation protocol (Figure 4G), hfPSC‐LCs were committed to SOX1^+^ neural precursor cells with highly ordered neural rosette around 5 days at a percentage over 80% (Figures 4G,H, S8H). The expression level of neural‐ectoderm regulators *SOX1* and *PAX6* were significantly increased during the induction of hfPSC‐LCs into neural precursor cells (Figure S8I). Ultimately, TUJ1^+^ neurons with stretched axon were obtained after the differentiation 7–10 days (Figure 4I). Collectively, hfPSC‐LCs have the differentiation potential of three‐germ‐layer.

To investigate the germline differentiation of hfPSC‐LCs, we established two distinct induction strategies for them: single‐cell aggregation of hfPSC‐LCs or intact clone of hfPSC‐LCs [33]. The induction of human primordial germ cell‐like cells (hPGCLCs) from hPSCs through incipient mesoderm‐like cells (iMeLCs) served as a positive control (Figure 4J) [51]. The hPGCLCs aggregates generated from intact hfPSC‐LCs colonies co‐expressed the early PGC markers SOX17 and TFAP2C/AP2γ around 6 days (Figure 4K) [71]. Flow cytometric analysis showed that ~50% of the cells induced from hfPSC‐LCs were EpCAM^+^/Integrin α6^+^ after 6‐day differentiation (Figure 4L,M) [51]. Meanwhile, hPGCLCs generated from single cells of hfPSC‐LCs also expressed NANOG and STELLA (Figure S8J,K). Several PGC markers, including *BLIMP1*, *TFAP2C*, *SOX17* and *NANOS3*, were significantly upregulated in hfPSC‐LCs derived‐hPGCLCs when compared to the undifferentiated hfPSC‐LCs (Figure S8L). These findings collectively show that hfPSC‐LCs can efficiently contribute to the germline cells.

### 3.4 Human Amnion‐Like Precursor Cells (hALPCs) Established From hfPSC‐LCs

**FIGURE 5:**
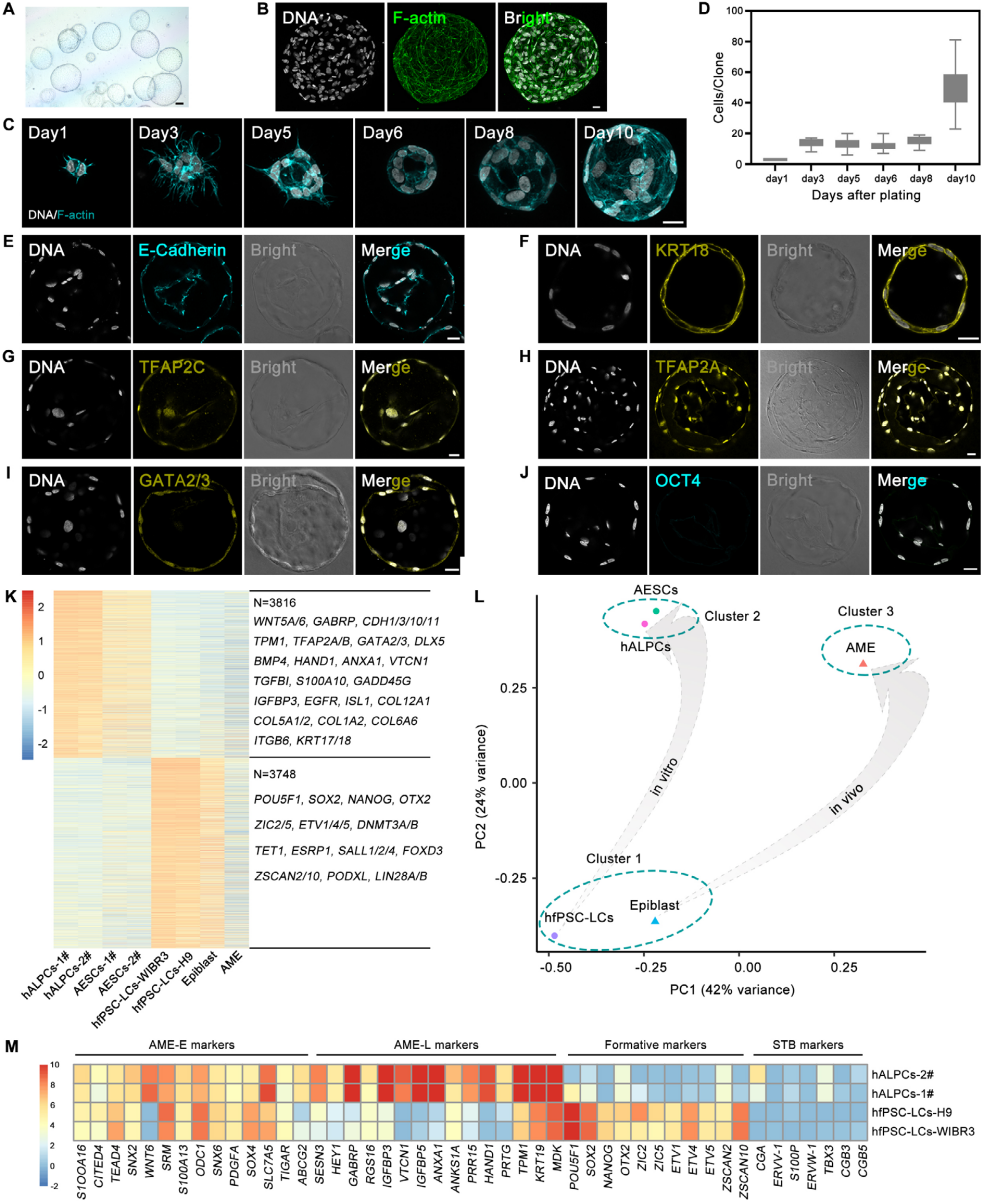
Human amnion‐like precursor cells (hALPCs) derived from hfPSC‐LCs. (A) The bright field of amnion‐like spheroids derived from hfPSC‐LCs. Scale bars, 50 μm. (B) The 3D reconstruction of human amnion‐like spheroids outlined by F‐Actin staining (green), DNA was stained with Hoechst 33342 (blue). Scale bar, 20 μm. (C) The transition of amnion‐like spheroids from single cells of hfPSC‐LCs recorded by the immunostaining of F‐Actin (red) at days 1, 3, 5, 6, 8 and 10, respectively. DNA was stained with Hoechst 33342 (blue). Scale bar, 20 μm. (D) Growth curves of amnion‐like spheroids recorded by calculating the number of nucleus in a single clone of human amnion spheres after 1, 3, 5, 6, 8 and 10 days of plating, respectively. (E–I) The immunostaining of the selected amnion lineage markers: E‐Cadherin, KRT18, TFAP2C, TFAP2A in combination with GATA2/3 in amnion‐like spheroids. DNA was stained with Hoechst 33342 (blue). Scale bars, 20 μm. (J) The immunofluorescent analysis of amnion‐like spheroids with antibody of OCT4/POU5F1 (cyan). DNA was stained by Hoechst 33342 staining (blue). Scale bar, 20 μm. (K) Heatmap of the highly expressed genes (N, number of genes) in hALPCs, amniotic epithelial stem cells (AESCs) [74], hfPSC‐LCs, Epiblast cells and AME [11] (fold change ≥ 1.5). Representative genes of different clusters were selected and listed in the right. (L) PCA plot of RNA‐seq data from in vitro (hfPSC‐LCs, hALPCs and AESCs) [74] and in vivo (Epiblast cells and AME) [11] samples. (M) Heatmap showing expression of the markers of AME‐E, AME‐L, formative pluripotency, and STB in hfPSC‐LCs and hfPSC‐LCs‐derived hALPCs, respectively.

Notably, ATAC signals are enriched on the genes related to the amnion specification (*GABRP*, *TPM1*, *TFAP2A*, *TFAP2B*, *GATA2*, *GATA3*, *DLX5*, *HAND1*, *VTCN1*, and *ISL1*) in hfPSC‐LCs, suggesting their potency for amniogenesis [72, 73]. To investigate this hypothesis, we assessed the differentiation potential of hfPSC‐LCs into amnion lineage through dual inhibition of the MAPK and ALK signalling pathways [38]. Bright‐field imaging and 3D resconstruction showed that hfPSC‐LCs transformed into large and saccular vacuoles (Figure 5A,B) when they were induced into amnion lineage. During the initial 5 days, prominent pseudopodia extended around the colonies and gradually retracted thereafter, coinciding with the emergence of a defined lumen (Figure 5C). Quantification of cell numbers of the spheres at days 1, 3, 5, 6, 8, and 10 further characterised the expansion of these structures (Figure 5D). Immunostaining showed the expression of E‐Cadherin, KERATIN 18 (KRT18), and key amnion transcription factors (TFAP2A, TFAP2C, and GATA2/3) in hfPSC‐LCs derived amnion spheres (Figure 5E–I). The epiblast marker POU5F1/OCT4 was undetectable in these amnion spheres induced from hfPSC‐LCs (Figure 5J), confirming their exit from pluripotency. Notably, we achieved the limited serial passaging of human amnion‐like cells (with a maximum passage up to P5) which we termed human amnion‐like precursor cells (hALPCs).

Subsequently, we investigated the transcriptome profiles of hALPCs using bulk RNA sequencing and compared the data with those of in vitro‐cultured human embryos (epiblast and amniotic epithelium, AME), amniotic epithelial stem cells (AESCs) isolated from the amniotic membrane of full‐term babies [11, 74] as well as hfPSC‐LCs. Hierarchical clustering revealed that hALPCs exhibited transcriptional similarities to AESCs but were distinct from both hfPSC‐LCs and in vivo epiblast cells. Differential expression analysis identified 3816 genes co‐upregulated in hALPCs and AESCs, while 3748 genes with elevated expression in hfPSC‐LCs and in vivo epiblast (Table S6) (fold change ≥ 1.5; *p*‐value < 0.05). A subset of amnion‐specific genes, including *WNT5A/6*, *GABRP*, *CDH1/3/10/11*, *TPM1*, *TFAP2A/B*, *GATA2/3*, *DLX5*, *BMP4*, *HAND1*, *ANXA1*, *VTCN1* as well as *TGFBI* were highly expressed in both hALPCs and AESCs (Figure 5K). Principal component analysis (PCA) of RNA‐seq datasets further confirmed the close relationship between hALPCs and AESCs, which clustered separately from hfPSC‐LCs and epiblast cells (Figure 5L). In addition, the hfPSC‐LCs derived hALPCs expressed markers of both early amniotic epithelium (AME‐E: *CITED4*, *TEAD4*, *SNX2*, *WNT6*, *SNX6*, *PDGFRA*, *SOX4*) and late amniotic epithelium (AME‐L: *GABRP*, *IGFBP3*, *VTCN1*, *IGFBP5*, *HAND1*, *TPM1*, *KRT19*, *MDK*). Whereas the pluripotency‐related genes and key syncytiotrophoblast (STB) regulators (including *CGA*, *ERVV‐1*, *S100P*, *ERVW‐1*, *TBX3*, *CGB3* and *CGB5*) were nearly undetectable (Figure 5M).

### 3.5 3D‐Matrigel Endows hfPSC‐LCs With Formative Features

Three‐dimensional (3D) cultures probably recapitulate in vivo cell–cell and cell‐matrix interactions, enabling cells to exhibit many unique and desirable characteristics under physiological conditions [75]. Matrigel, a basement membrane matrix extracted from the Engelbreth‐Holm‐Swarm (EHS) mouse sarcoma, is widely employed for 3D cell cultures [76, 77, 78]. Matrigel probably provides a complex protein mixture required for optimal cell growth and stem cell pluripotency maintenance [79], making it an excellent substitute for in vivo physiological condition.

**FIGURE 6:**
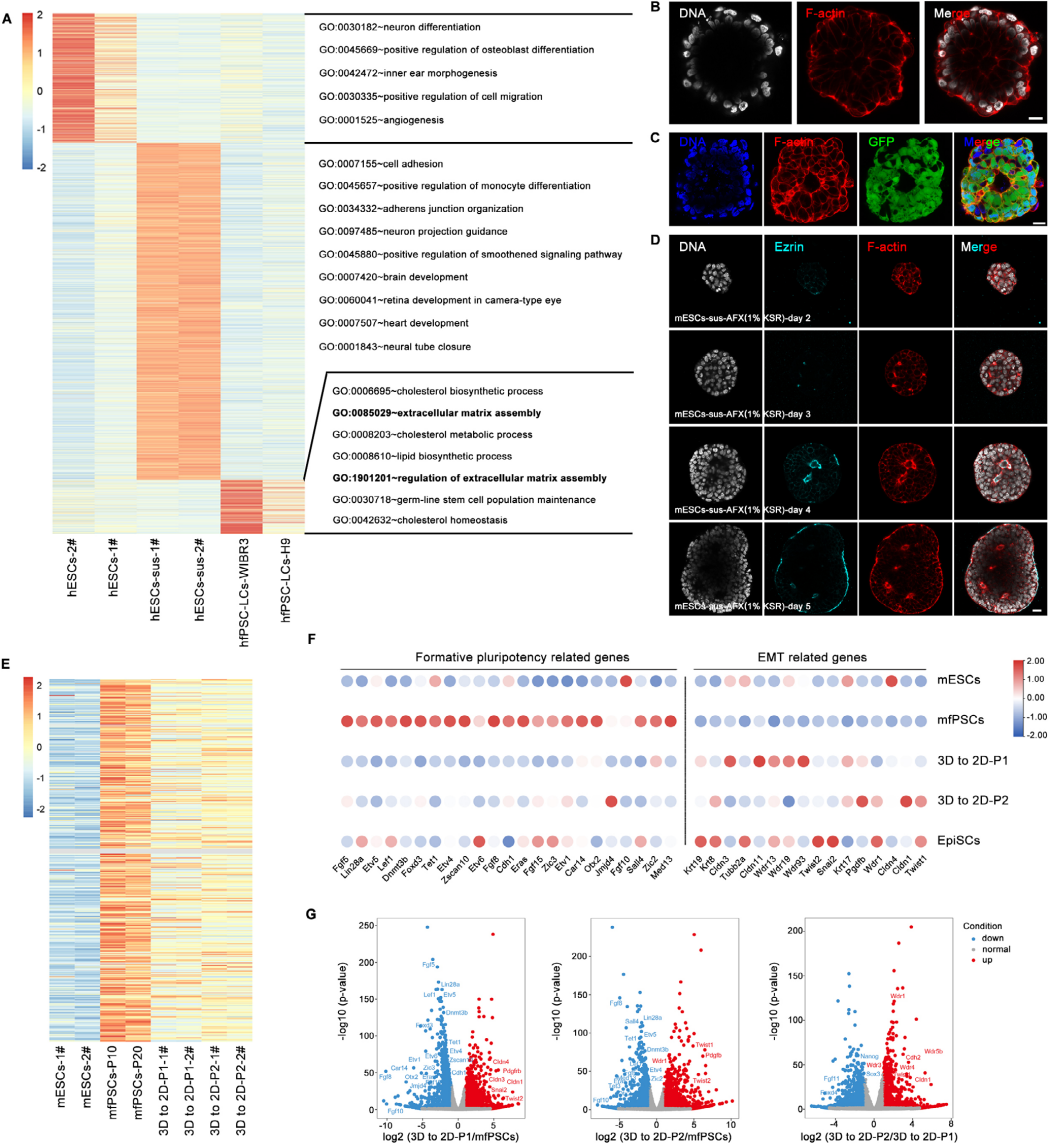
3D‐Matrigel endowed hfPSC‐LCs and fPSCs with formative pluripotency. (A) Heatmap and enrichment of GO terms of genes upregulated in conventional hESCs (E8), suspension cultured hESCs (E8), as well as hfPSC‐LCs (E8 + IWP2) (fold change ≥ 1.5), respectively. (B) Immunofluorescence assay of suspension cultured hESCs [80] stained with the Phalloidin (F‐Actin, red) and Hoechst 33342 (white). Scale bar, 20 μm. (C) Colonies aggregated from single cells of GFP+ and GFP‐ hESCs in E8 medium and in suspension manner reported by Zongyong Ai [80]. DNA was stained with Hoechst 33342 (blue). Scale bar, 20 μm. (D) Immunostaining of the mouse spheroids cultured in suspension manner using the mouse fPSCs medium from day 2 to day 5. Ezrin was employed to indicate the cavity in the spheroids. DNA was stained with Hoechst 33342 (blue). Scale bar, 20 μm. (E) The heatmap of formative gene identified previously [33] in mESCs, mouse fPSCs at P10/20 and mouse fPSCs cultured in Matrigel coated dishes for 1 and 2 passages (3D to 2D‐P1, 3D to 2D‐P2, 4 days/passage). (F) The expression of selected formative markers and EMT related genes in mESCs, mouse fPSCs (mfPSCs), mouse fPSCs cultured in Matrigel coated dishes for 1 and 2 passages (3D to 2D‐P1, 3D to 2D‐P2, 4 days/passage) and EpiSCs detected by RNA‐seq. (G) The volcano plot showing genes in mouse fPSCs versus mouse fPSCs cultured in Matrigel coated dishes for 1 and 2 passages (3D to 2D‐P1, 3D to 2D‐P2, 4 days/passage), and mouse fPSCs cultured in Matrigel coated dishes for 1 passage versus 2 passages (3D to 2D‐P1, 3D to 2D‐P2, 4 days/passage). Gene symbols are shown for selected EMT related genes (red) and formative pluripotency (blue) genes. The RNA‐seq data for mESCs, fPSCs as well as EpiSCs were obtained from our previously published datasets [33].

Matrigel provides both 3D structural scaffolding and essential extracellular matrix (ECM) components. We further investigated the function of Matrigel in formative cell formation within the 3D culture system. To minimise dish‐related effects on adherent cell cultures, we employed two control groups in our experiments: hESCs maintained in suspension manner [80] and conventional cultured hESCs (cultured on Matrigel coated dish) [81]. Transcriptional profiling revealed distinct gene expression patterns among these cell types. Specifically, hfPSC‐LCs exhibited upregulation of genes associated with extracellular matrix assembly and its regulation, whereas both conventional and suspension‐cultured hESCs showed elevated expression of genes linked to multi‐organs and tissues development (fold change ≥ 1.5; *p*‐value < 0.05) (Figure 6A) [80]. On the other hand, hfPSC‐LCs underwent metabolic reprogramming characterised by enhanced cholesterol and lipid biosynthesis pathways. This shift is likely attributable to the 3D culture environment and appears to play a pivotal role in establishing and sustaining formative pluripotency (Figure 6A).

Morphological analysis showed distinct colony architectures: suspension‐cultured hESCs failed to form characteristic rosette structure with a single apical lumen, instead developing into disorganised cell clusters (Figure 6B). Unlike the single‐cell clonogenicity of hfPSC‐LCs, suspension‐cultured hESCs exhibited dependence on cellular aggregation for colony formation, as confirmed by GFP^+^/GFP^−^ co‐culture tracking experiments (Figure 6C). When single cells of GFP^+^ and GFP^−^‐hESCs were mixed and cultured in low‐attachment 24‐well plates, the resulting colonies consistently contained both GFP^+^ and GFP^−^ cells (mosaic‐like colonies) (Figure 6C). These findings provide evidence that hESCs colonies in suspension culture originate through the aggregation of multiple cells rather than from single‐cell expansion (Figure 6C and Figure S2C). This phenotype was consistently observed in mouse fPSCs, which exhibited comparable loss of apical‐basal polarity and developed into irregular colony morphology under suspension culture conditions (Figure 6D). During prolonged culture (days 2–5), we observed progressive clone expansion accompanied by the emergence of multiple lumens within the disorganised cellular architecture (Figure 6D).

Transcriptomic analysis between mouse fPSCs and mouse fPSCs undergoing 2D adaptation on Matrigel‐coated dishes for 1 and 2 passages (3D to 2D‐P1 and 3D to 2D‐P2) revealed significant changes of formative gene expression (Figure 6E). Compared to 3D‐cultured mouse fPSCs, formative pluripotency master genes (*Fgf5*, *Lin28a*, *Etv5*, *Dnmt3b*, *Etv4*, *Zscan10*, *Otx2*, and *Zic2*) exhibited significant down‐regulation, while epithelial‐mesenchymal transition (EMT)‐related genes (*Twist2*, *Snai2*, *Pdgfb*, and *Twist1*) were up‐regulated (Figure 6F) in 2D‐adapted mouse fPSCs (mfPSCs). Volcano plot analysis further confirmed these transcriptional changes (Figure 6G). These findings collectively demonstrate that 3D‐Matrigel deprivation induces coordinated morphological and molecular changes in both mouse fPSCs and hfPSC‐LCs, probably reflecting partial loss of formative pluripotency characteristics in 2D condition.

To evaluate the functional impact of 2D adaptation of mouse fPSCs, we performed the differentiation assays for them. Neural induction experiments revealed significantly reduced efficiency in generating neural precursor cells (< 60% Sox1‐GFP^+^ cells) from 2D‐adapted mouse fPSCs compared to 3D‐cultured mouse fPSCs (Figure S9A,B). Strikingly, cardiomyocyte differentiation protocols failed to produce beating clusters from 2D‐adapted mouse fPSCs after 5 days of induction, with cultures predominantly containing apoptotic cells (Figure S9C). Furthermore, attempts to generate mouse primordial germ cell‐like cells (mPGCLCs) from 2D‐adapted mouse fPSCs using standard protocols barely yielded BV^+^/SC^+^ aggregates (Figure S9D). Collectively, these findings demonstrate severe impairment of multi‐lineage differentiation potential when mouse fPSCs were cultured in 2D conditions.

## 4 Discussion

Naïve and primed pluripotenct stem cells probably recapitulate two ends of the pluripotency spectrum during mammalian early development. Both human and mouse naïve ESCs represent the uncommitted state, probably requiring systematic dismantling of naïve‐specific regulatory networks through a precisely orchestrated capacitation process for the differentiation into primed state. This transitional phase of differentiation probably involves sequential activation of transcriptional programs to establish a formative state prior to lineage commitment. Mouse EpiSCs and primed hESCs are deemed as fate‐biased and harboured a certain proportion of fate‐determined cells. Currently, several intermediate/formative pluripotent cell lines can be captured in both humans and mice [27, 29, 31, 33, 34, 68, 82, 83]. Formative pluripotent cells are intermediate state and probably are freed from naïve‐phase constraints and lineage biases, consistent with their competence for homogeneous differentiation into embryonic lineages. Thus, formative pluripotent cells are highly promising candidates for regenerative medicine applications.

**FIGURE 7:**
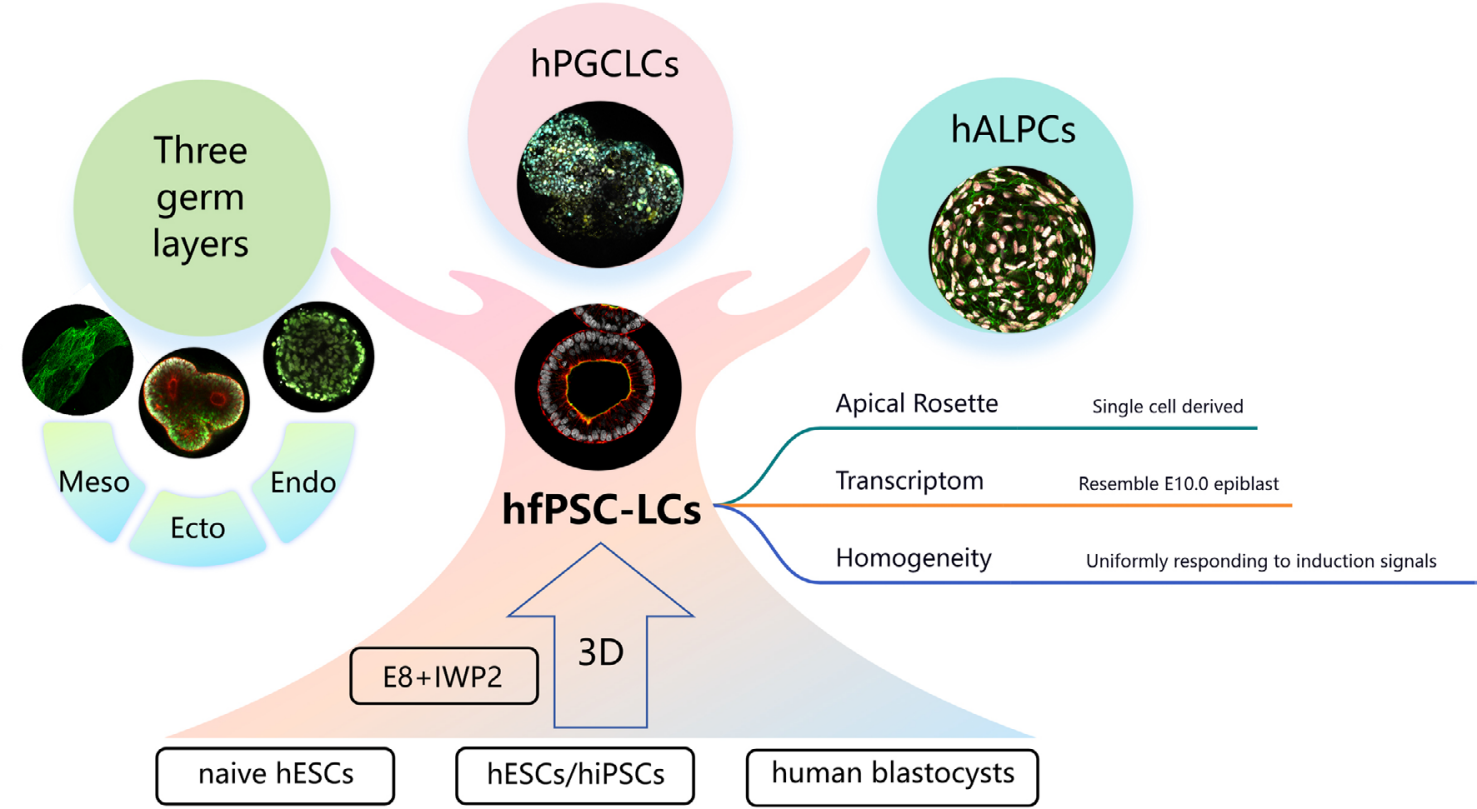
A model illustrating the establishment of hfPSC‐LCs. Human formative pluripotent stem cells (hfPSC‐LCs) can be robustly derived from multiple sources, including naïve human embryonic stem cells (hESCs), conventional hESCs, human induced pluripotent stem cells (hiPSCs), and human blastocysts. When cultured as single cells in 3D Matrigel, hPSCs spontaneously self‐organise into apical‐basal polarised rosette structures. Transcriptomic profiling demonstrates that hfPSC‐LCs transcriptionally recapitulate the post‐implantation epiblast at ~10 days post‐fertilisation, exhibiting enhanced population homogeneity relative to other pluripotent states. Strikingly, these single‐layer rosette colonies display synchronised and highly efficient differentiation capacity, readily generating all three embryonic germ layers, human primordial germ cell‐like cells (hPGCLCs), and human amnion‐like precursor cells (hALPCs) in response to lineage‐specific cues.

In this study, we developed a biomimetic 3D culture system that probably recapitulates peri‐implantation embryonic microenvironments, enabling a robust establishment of self‐renewing hfPSC‐LCs. These cells exhibit unique molecular signatures that clearly distinguish them from previously characterised hXPSCs and hFS‐like cells [29, 34], thereby representing a novel formative‐like pluripotency stage (Figure 7). Developmental plasticity analysis revealed that hfPSC‐LCs possess tri‐lineage (ectoderm/mesoderm/endoderm), hPGCLCs, and amnion‐like cells differentiation competence, positioning them as a versatile platform for organoid modelling of human early embryogenesis. Similar to mouse fPSCs, hfPSC‐LCs directly differentiate into hPGCLCs through single‐cell aggregation or intact clonal units, bypassing the incipient mesoderm‐like cells (iMeLCs) transition required for hPGCLCs induction from primed hiPSCs [51]. The hPGCLCs derived from hfPSC‐LCs via cloning aggregation offer a straightforward and user‐friendly approach while reducing digestion‐induced cell death.

Previous studies have shown that under the AME induction system, differentiation initiated from primed hESCs yields a cell population comprising approximately 80% STB cells [38], suggesting that STB formation is a major byproduct of amniotic cell derivation in these systems. Additionally, a small subset of cells in the partially primed hESCs differentiation system maintains pluripotency and fails to fully differentiate into amniotic lineage [38]. In this study, we find that the hALPCs induced from hfPSC‐LCs expressed both AME‐E and AME‐L markers. Furthermore, STB and pluripotent markers were barely detected in the hALPCs induced from hfPSC‐LCs. Thus, our findings suggest that the hALPCs derived from hfPSC‐LCs probably recapitulate the molecular signatures of bona fide AESCs, providing a robust in vitro platform for modelling human amniogenesis.

Our findings further demonstrate that deprivation of 3D culture environments significantly compromises the differentiation capacity of fPSCs. The hfPSC‐LCs exhibit upregulated extracellular matrix (ECM) assembly regulators compared to both adherent and suspension‐cultured hESCs, suggesting active ECM remodelling is essential for formative pluripotency establishment or maintenance. Matrigel, derived from EHS mouse cells, is composed of a diverse and abundant mixture of ECM proteins that may mimic the functions of the natural basement membrane [83]. Integrins probably present in Matrigel engage with receptors on hfPSC‐LCs, transducing extracellular signals intracellularly. This cascade may facilitate the establishment and maintenance of cell polarity and formative pluripotency, consistent with the failure of β1‐integrin knockout mESCs to form polarised rosettes [84]. In addition, compared with those in 2D, the formative cells cultured in 3D condition predominantly adopt a monolayer‐like cellular organisation, exhibiting more comparable cell–cell interactions and cell–extracellular matrix interactions. This architectural uniformity is expected to facilitate more consistent intercellular signal transduction and cell–cell communication, thereby establishing a more homogeneous growth microenvironment within each clone. Thus, three‐dimensional structure may provide fPSCs with a physically uniform culture environment characterised by consistent stiffness and confined adhesiveness, thereby promoting cellular homogeneity [85]. How the 3D‐Matrigel environment contributes to the establishment and maintenance of formative cells warrants further study.

## Author Contributions

X.W. designed and performed the major experiments, analysed the data, and wrote the original manuscript; Q.W. performed the analysis of bioinformatics. Y.W. performed scRNA‐seq analysis and prepared the ATAC sequencing library. C.G. and M.F.L. established the *SNAI1*‐mCherry knock‐in cell line. Y.F., X.K., L.L., Z.G., M.H., and J.L. provided the human blastocysts and analysed the data. L.L. initiated, organised, and designed the study, analysed the data, and finalised the manuscript with the comments from all authors.

## Funding

This study was supported by the National Key R&D Program of China (2024YFA1106900, 2021YFC2700300, 2024YFA1106902, 2024YFC2706604) and the National Natural Science Foundation of China (32200649, 32570941).

## Ethics Statement

This study was approved by the Medical Ethics Committee of the Third Affiliated Hospital of Guangzhou Medical University. All donated embryos were cryopreserved surplus specimens from couples who had successfully undergone in vitro fertilisation (IVF) treatment. Prior to donation, all couples provided written informed consent for the voluntary use of their surplus embryos in human embryo development research conducted at the Third Affiliated Hospital of Guangzhou Medical University.

## Conflicts of Interest

The authors declare no conflicts of interest.

## Supporting information


**Data S1:** Supplementary Figures.

**Table S1:** Primers used in this study.

**Table S2:** Genes highly expressed in naive hESCs, hfPSC‐LCs, and primed hESCs.

**Table S3:** Genes highly expressed in hXPSCs, hFS‐like cells, and hfPSC‐LCs.

**Table S4:** Genes exhibiting both active transcription (TPM ≥ 5) and accessible promoters (RPKM ≥ 3) in hfPSC‐LCs.

**Table S5:** Bivalent gene list.

**Table S6:** Genes highly expressed in hfPSC‐LCs derived hALPCs.

**Movie S1:** The rhythmic contractions of cardiac colonies derived from hfPSC‐LCs.

**Movie S2:** The recording of calcium transient signals in cardiomyocytes derived from hfPSC‐LCs.

## Data Availability

The RNA‐seq, scRNA‐seq, ATAC‐seq and WGBS datasets in this study have been deposited to NCBI with accession number: GSE298432.
