# Supplementary material for: Establishment of Human Formative Pluripotent Stem Cell‐Like Cells Exhibiting Amniotic Differentiation Potentials
Source: Cell Prolif. 2026 Mar 25;59(8):e70193. doi: 10.1111/cpr.70193 (PMC13325813; doi:10.1111/cpr.70193)
Supplement: Supplementary file 1 [file CPR-59-e70193-s008.pdf]

Figure S1

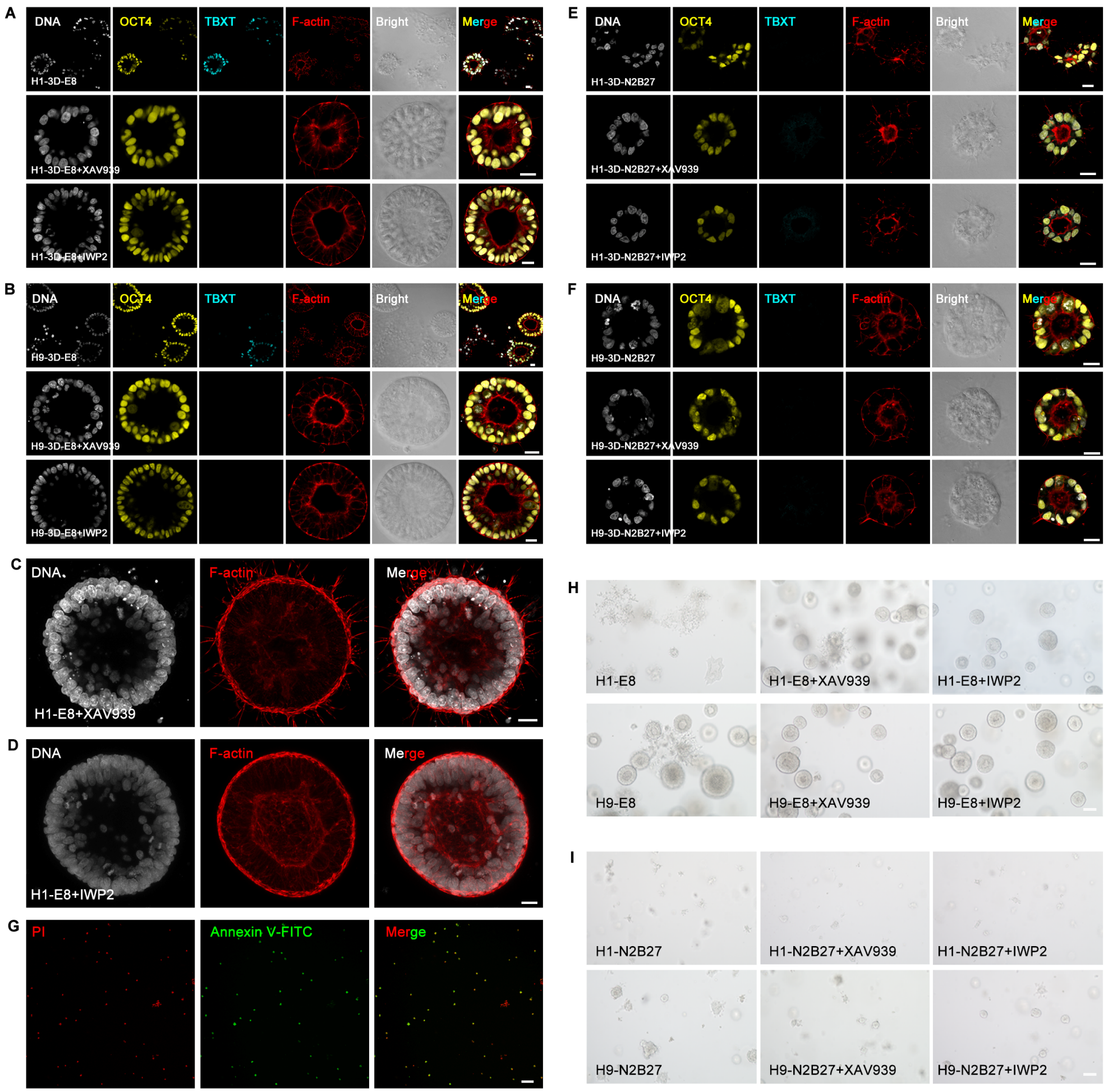

FIGURE S1 | Optimization the culture system for human Rosette clones. (A-B) The immunostaining of OCT4/POU5F1 (yellow) and TBXT (cyan) in hESCs (H1 and H9, respectively) cultured in 3D-Matrigel supplemented with the medium of E8/ E8+XAV939 (5 μM)/ E8+IWP2 (2 μM). Apical lumen was labeled by Phalloidin staining (F-actin, red). DNA was stained with Hoechst 33342 (white). Scale bars, 20 μm. (C-D) Three-dimensional reconstruction of hESCs (H1) cultured in 3D-Matrigel with E8+XAV939 (5 μM) (C) and E8+IWP2 (2 μM) (D), respectively. Apical lumen and the pseudopodium was labeled by Phalloidin staining (F-actin, red). DNA was labeled by Hoechst 33342 (white). Scale bars, 20 μm. (E-F) The immunostaining of OCT4/POU5F1 (yellow) and TBXT (cyan) in hESCs (H1 and H9, respectively) cultured in 3D-Matrigel supplemented with the medium of N2B27/ N2B27+XAV939 (5 μM)/ N2B27+IWP2 (2 μM). Apical lumen was labeled by Phalloidin staining (F-actin, red). DNA was stained with Hoechst 33342 (white). Scale bars, 20 μm. (G). The apoptotic (Annexin V-FITC, green) and necrotic cells (Annexin V-FITC+PI, green+red) indicated by Annexin V-FITC Apoptosis Detection Kit. Scale bar, 100 μm. (H-I) The bright field of hESCs (H1 and H9) cultured in 3D-Matrigel supplemented with the medium of E8/ E8+XAV939 (5 μM)/ E8+IWP2 (2 μM) (H) and N2B27/ N2B27+XAV939 (5 μM)/ N2B27+IWP2 (2 μM) (I). These images were captured by microscopy at day 5. Scale bars, 50 μm.

Figure S2

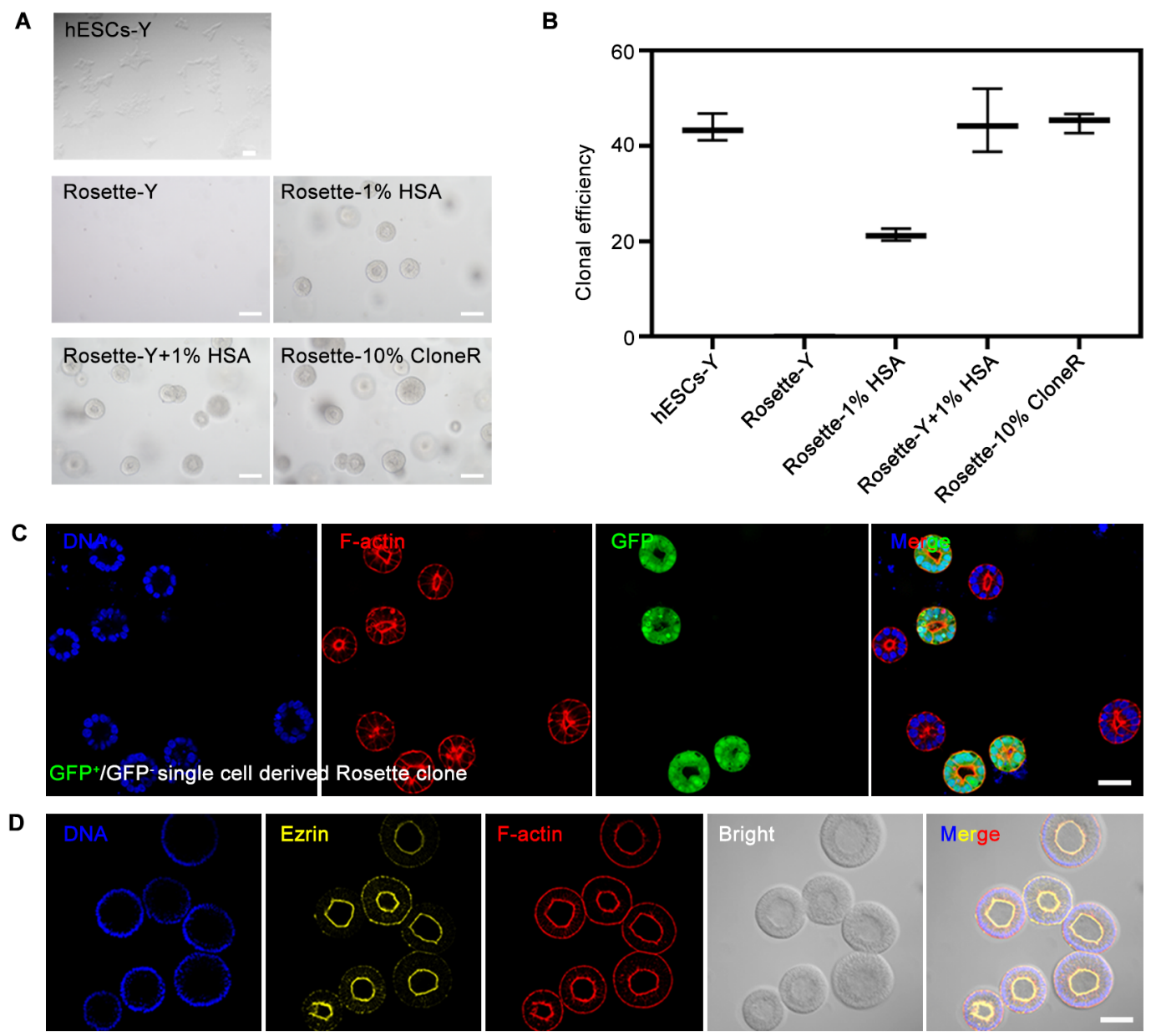

FIGURE S2 | Single cell propagation of hfPSC-LCs. (A)The bright-field (BF) image showing typical colony morphology of hESCs (H1, hESCs-Y) grown in E8 medium and Rosette clones obtained in E8+IWP2+Y27632 (Rosette-Y), E8+IWP2+1% HSA (Rosette-1% HSA), E8+IWP2+Y27632+1% HSA (Rosette-Y+1% HSA), and E8+IWP2+10% CloneR (Rosette-10% CloneR) medium. Scale bars, 100 μm. (B) Single-cell clonal efficiencies of hESCs (H1, hESCs+Y) and Rosette clones obtained from E8+IWP2+Y27632 (Rosette-Y), E8+IWP2+1% HSA (Rosette-1% HSA), E8+IWP2+1% HSA+Y27632 (Rosette-Y+1% HSA), and E8+IWP2+10% CloneR (Rosette-10% CloneR) medium. Data were represented as means ± SEM (n = 3). (C) GFP^+ and GFP^- Rosette clones derived from GFP^+ and GFP^- hESCs (H9) single cell, respectively. F-actin was stained with Phalloidin (red). Scale bar, 50 μm. (D) The morphology of the hfPSC-LCs established from conventional hESCs (H9). Apical lumen was labeled by Ezrin (yellow). Phalloidin labeled with Alex Fluor 546 was used to detect F-actin (red). DNA was stained with Hoechst 33342 (blue). Scale bar, 100 μm.

Figure S3

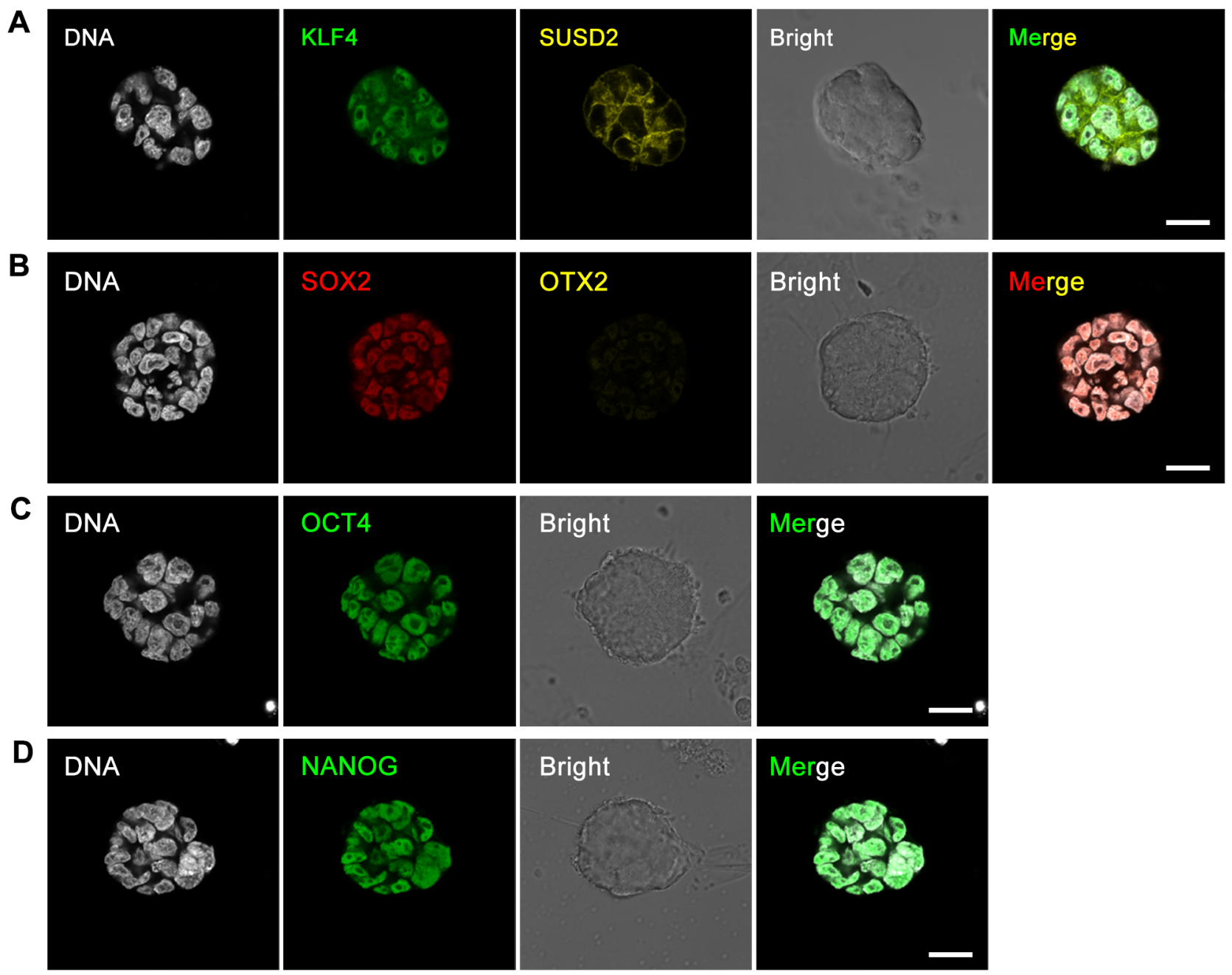

FIGURE S3 | Characterization of naïve hESCs (5iLA, WIBR3). (A-D) Immunostaining of the expression of KLF4 (green), SUSD2 (yellow), SOX2 (red),OTX2 (yellow), OCT4/POU5F1 (green), and NANOG (green) in naïve hESCs. DNA was stained with Hoechst 33342 (white). The results were captured with confocal laser scanning microscope with ×40 water objective. Scale bars, 20 μm.

Figure S4

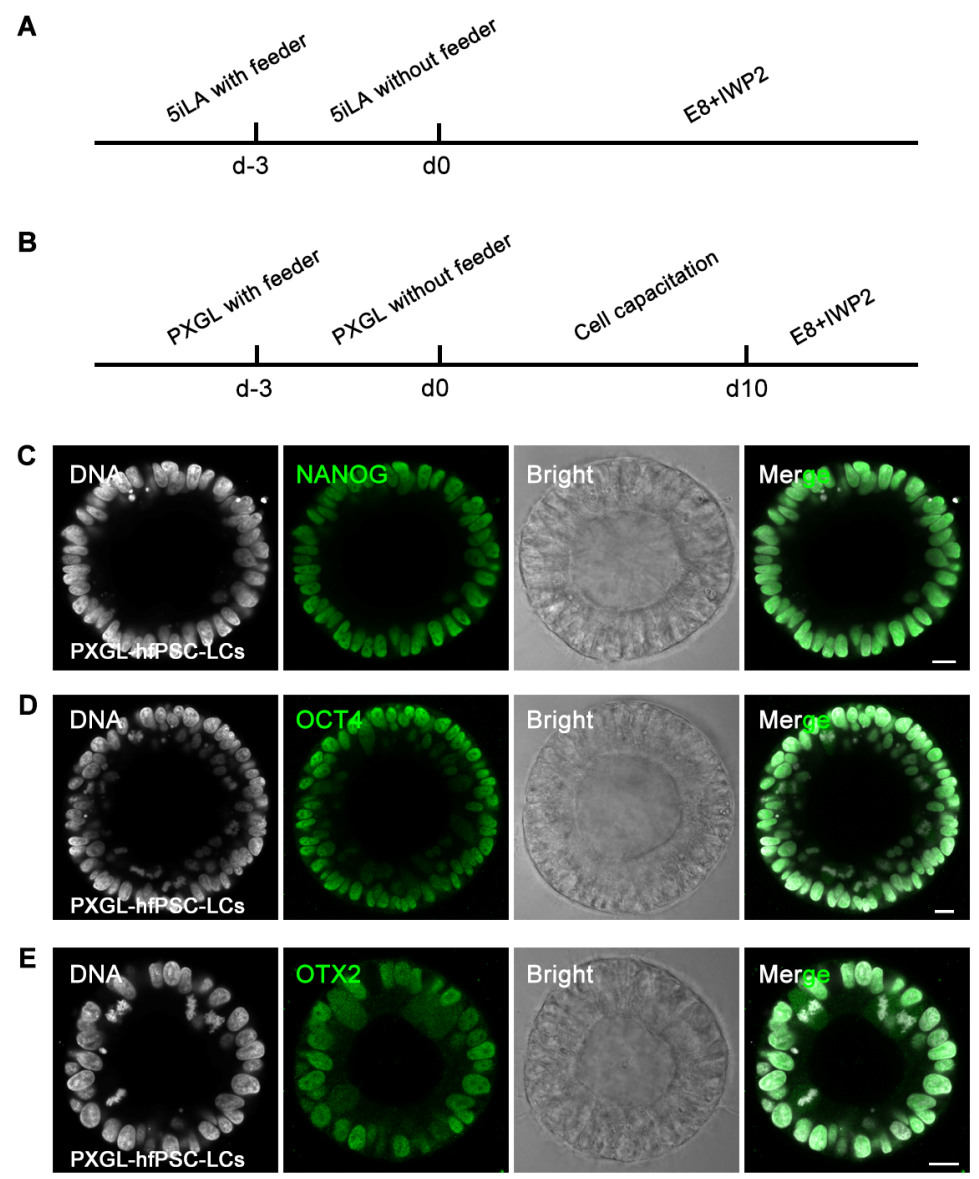

FIGURE S4 | The establishment of hfPSC-LCs from naive hESCs. (A and B) The flowchart of the transformation from 5iLA naive hESCs (A) and PXGL naive hESCs (B) to hfPSC-LCs, respectively. (C-E) The immunofluorescence analysis of NANOG (green), OCT4/POU5f1 (green), and OTX2 (green) in hfPSC-LCs established from PXGL-naive hESCs. DNA was labeled with Hoechst 33342 (white). The images were captured with confocal laser scanning microscope with ×40 water objective. Scale bars, 20 μm.

Figure S5

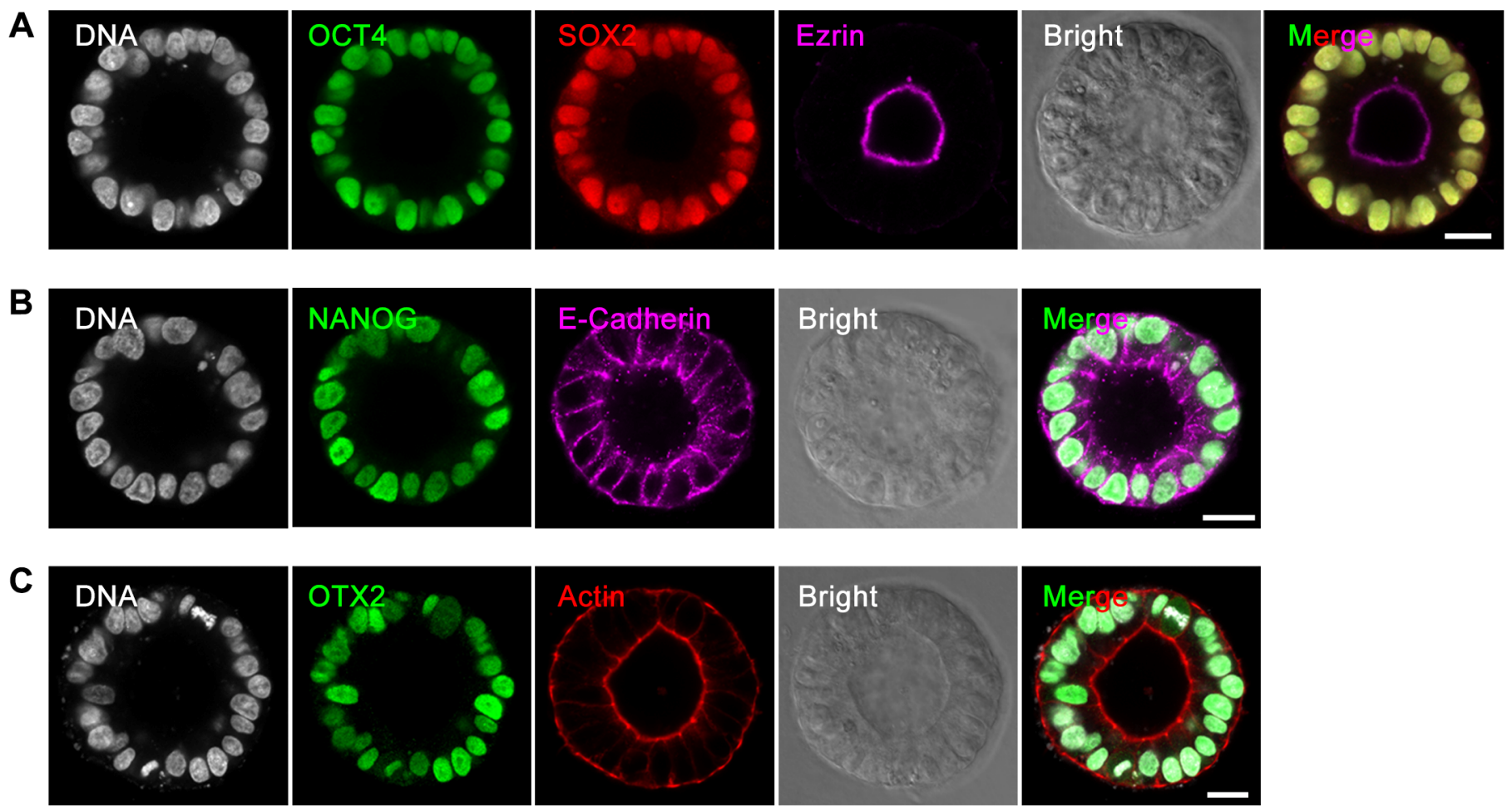

FIGURE S5 | The establishment of hfPSC-LCs from single cells of hiPSCs. (A-C) The immunofluorescent analysis of OCT4 (green), SOX2 (red), EZRIN (magenta), NANOG (green), E-Cadherin (magenta) as well as OTX2 (green) in hiPSCs derived hfPSC-LCs. F-actin was stained with Phalloidin (red). The apical lumen was labeled by Ezrin and F-Actin staining. Nuclei were labeled by Hoechst 33342 (white). The images were captured with confocal laser scanning microscope with ×40 water objective. Scale bars, 20 μm.

Figure S6

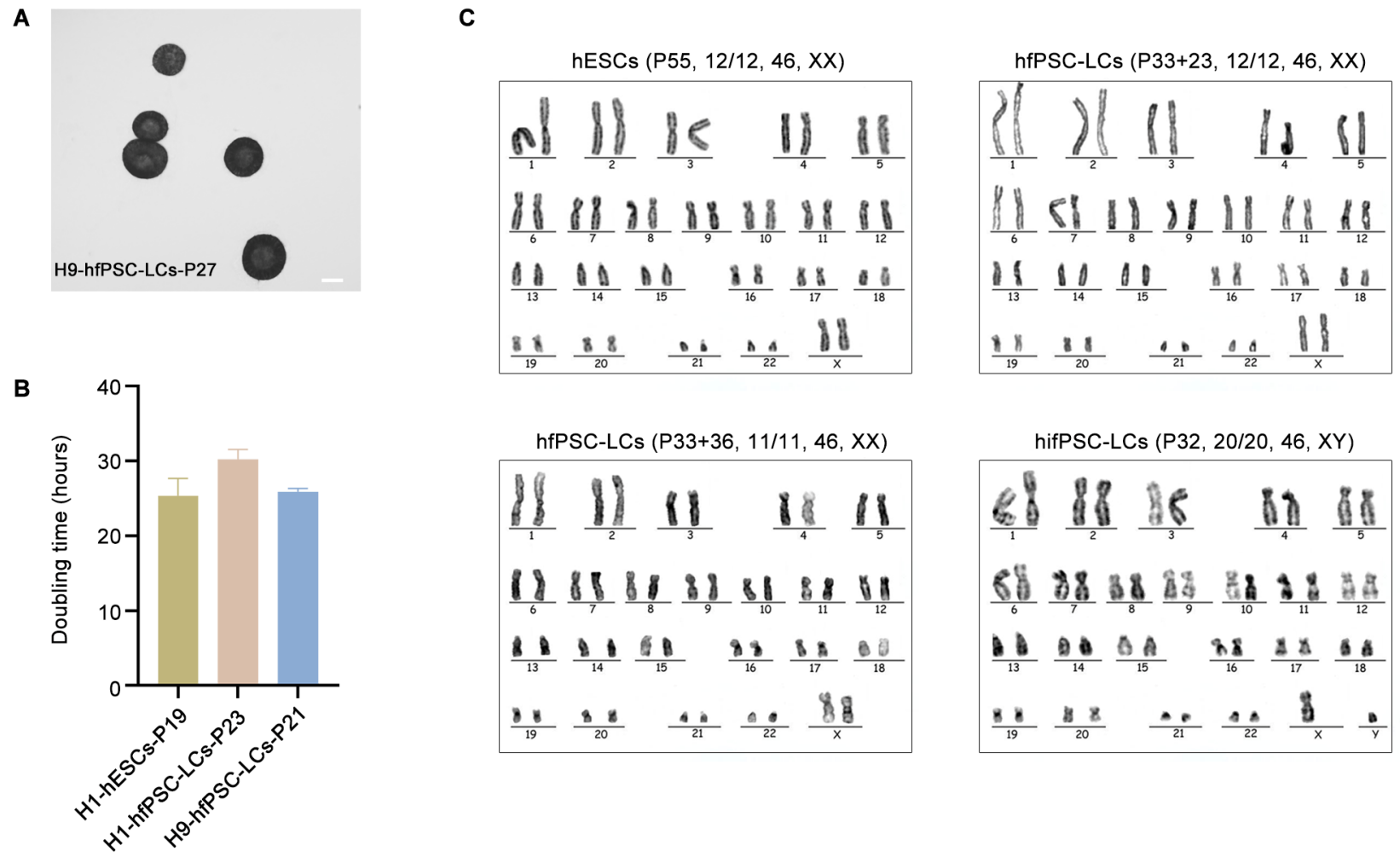

FIGURE S6 | The detailed features of hfPSC-LCs. (A) Alkaline phosphatase staining was performed for H9 derived hfPSC-LCs (H9-hfPSC-LCs) at passage 27. After staining, the images were captured with microscopy. Scale bar, 50 μm. (B) Bar graph showing the doubling time of H1-hESCs at passage 19, H1 derived hfPSC-LCs (H1-hfPSC-LCs) at passage 23, and H9 derived hfPSC-LCs (H9-hfPSC-LCs) at passages 21 using online statistics (https://www.doubling-time.com/compute.php?lang=en). (C) The karyotype analysis was performed of H9-hESCs (P55, 12/12), H9 derived hfPSC-LCs (P33+23,12/12; P33+36, 11/11), and hifPSC-LCs at passage 32 (20/20).

Figure S7

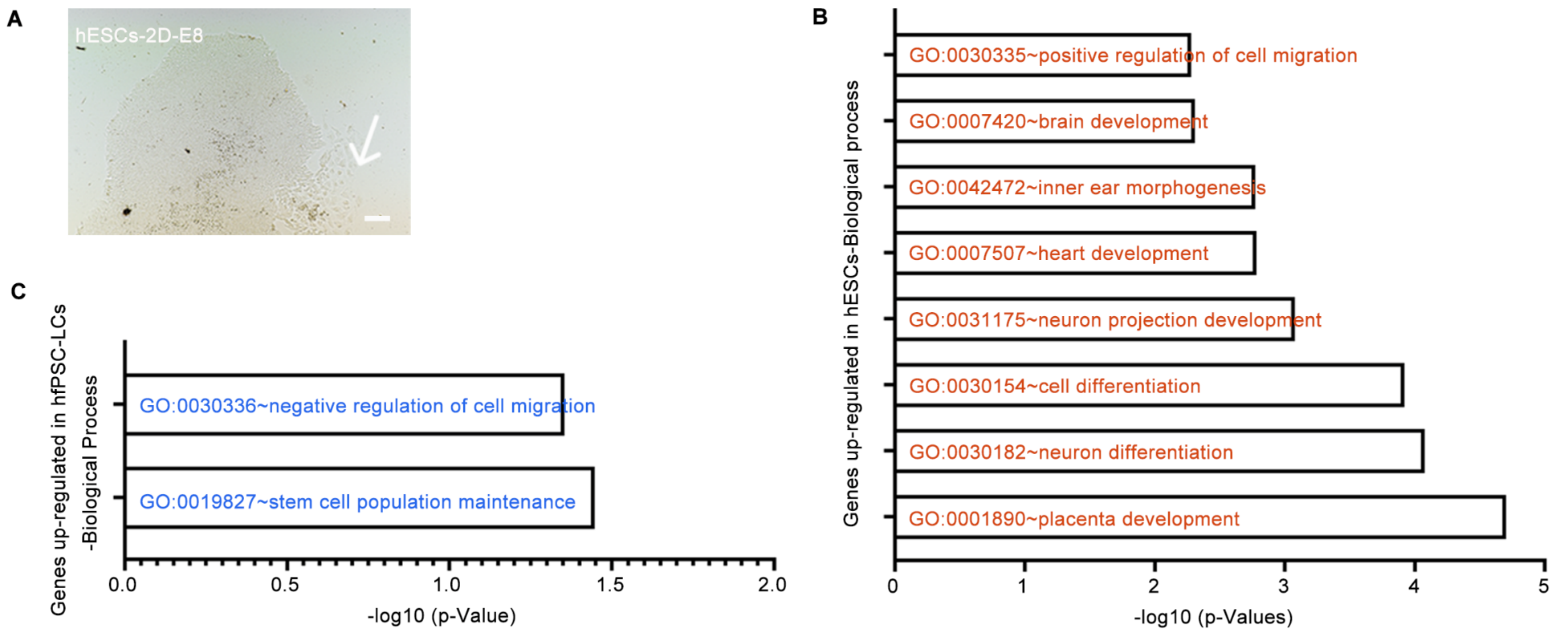

FIGURE S7 | The comparison between hESCs-E8 and hfPSC-LCs. (A) The bright field of hESCs (H9) cultured in E8 medium on Matrigel (Matrigel was diluted with DMEM/F12 in a ratio of 1:100) coated 6-well cell culture plate. The white arrowhead indicated the auto-differentiated cells. Scale bar, 50 μm. (B and C) GO analysis of the Biological Processes (BP) related to genes upregulated in hESCs (B) and in hfPSC-LCs (C), respectively. DAVID online analysis was used for GO enrichment analysis (https://davidbioinformatics.nih.gov/).

Figure S8

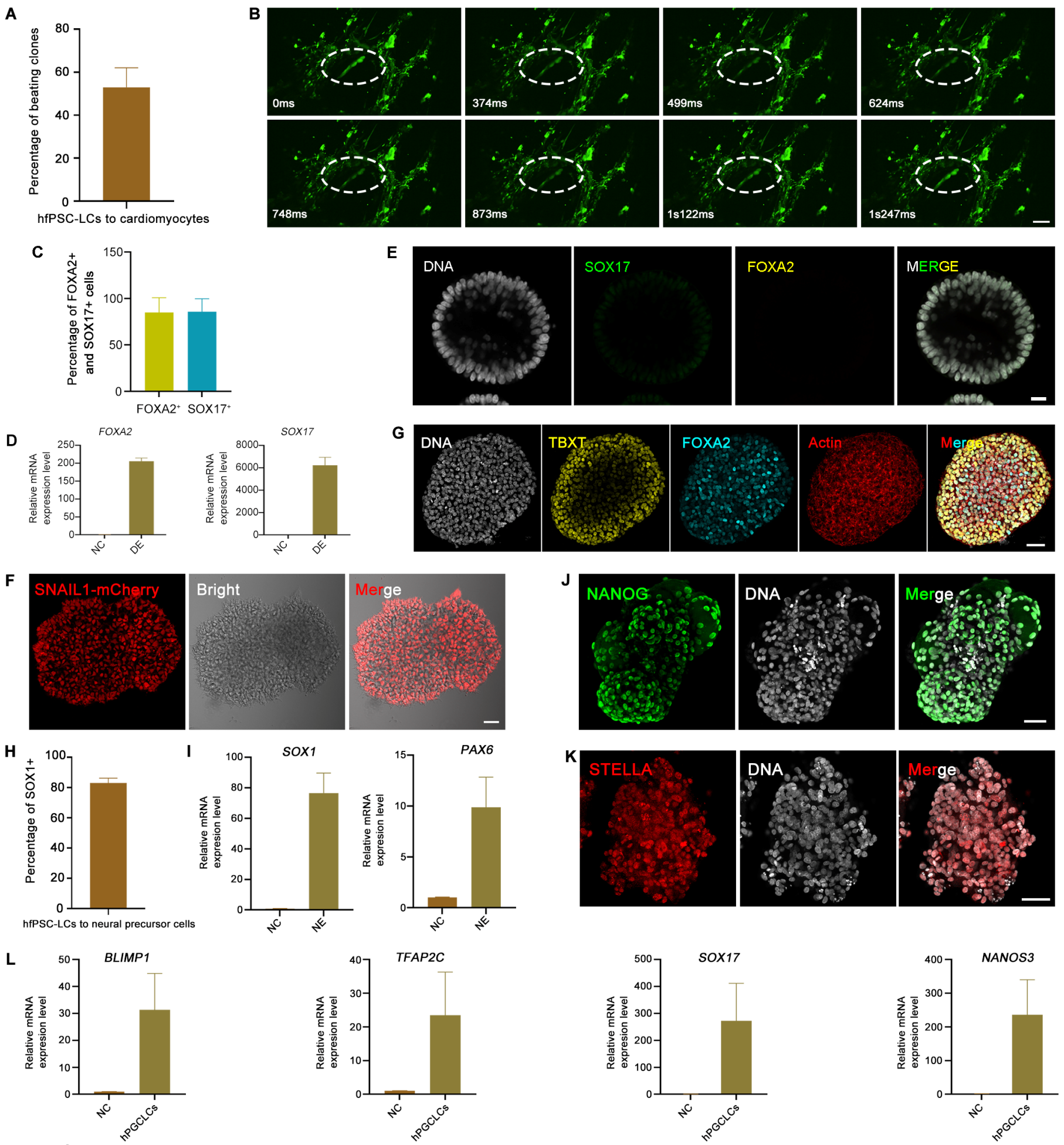

FIGURE S8 | The differentiation capacity of hfPSC-LCs. (A) The proportion of beating cardiomyocyte-like clones induced from hfPSC-LCs. All experiments were repeated at least three times. Error bars represented SEM. (B) Time-lapse recording of Ca^{2+} dynamics in cardiomyocyte-like cells derived from hfPSC-LCs following 10-day differentiation. Scale bar, 50 μm. (C) The percentage of FOXA2 and SOX17 positive cells were calculated in definitive endoderm cells induced from hfPSC-LCs. All experiments were repeated at least three times. Error bars represented SEM. (D) Gene expression of definitive endoderm induction from hfPSC-LCs and the undifferentiated hfPSC-LCs served as negative control. (E) The immuno-fluorescent analysis of definitive endoderm markers, SOX17 (green) and FOXA2 (yellow) in hfPSC-LCs. DNA was stained by Hoechst 33342 staining. Scale bar, 40 μm. (F) The expression of mCherry in irregular clones derived from *SNAIL1*-mCherry hfPSC-LCs after differentiation for 48 hours. Scale bar, 50 μm. (G) The immunofluorescence analysis of TBXT (yellow), FOXA2 (cyan), and Actin (red) after 48 hours differentiation of hfPSC-LCs. (H) The percentage of SOX1 positive cells differentiated from hfPSC-LCs. All experiments were repeated at least three times. Error bar represented SEM. (I) Gene expression of neural ectoderm (NE) cells induction from hfPSC-LCs and the undifferentiated hfPSC-LCs served as negative control. (J and K) The immunostaining of NANOG (green) and STELLA (red) in hPGCLCs aggregates from single cells of hfPSC-LCs. DNA was stained by Hoechst 33342 staining. Scale bars, 50 μm. (L) The relative expression of *BLIMP1*, *TFAP2C*, *SOX17* and *NANOS3* in hPGCLCs differentiated from hfPSC-LCs. The undifferentiated hfPSC-LCs served as negative control.

Figure S9

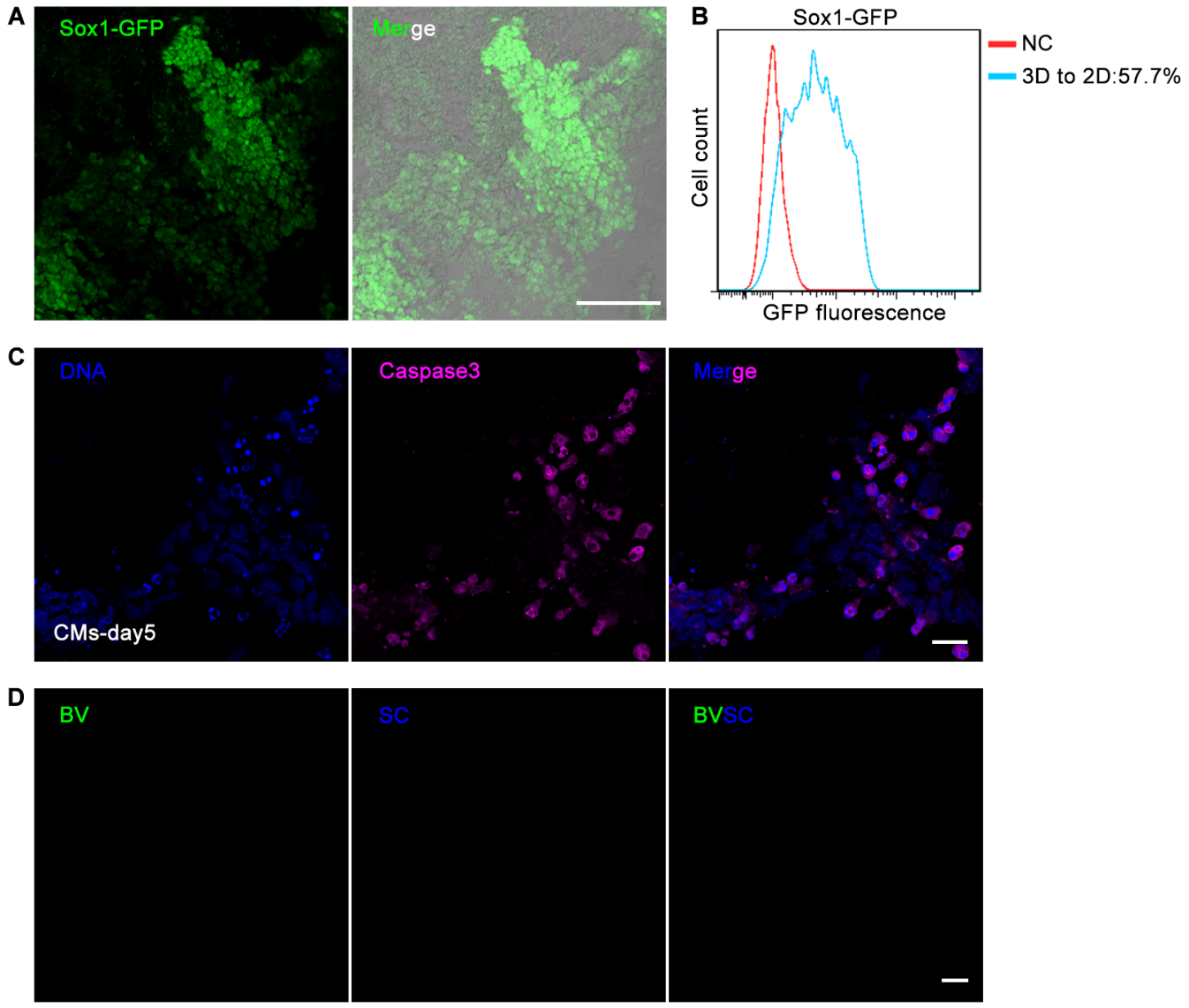

FIGURE S9 | Three dimensional environments ensure mfPSCs with better differentiation capacity. (A) Single cells of mfPSCs (46C:Sox1-GFP, P23) were cultured on Matrigel coated dish for 2 days and then differentiated to neural lineage after another 2 days induction, Sox1-GFP⁺ cells were shown. Scale bar, 100 μm. (B) FACS analysis result was shown in left panel (NC, undifferentiated Sox1-GFP mfPSCs). (C) CMTI-1-mfPSCs cultured on Matrigel coated dish for 4 days and then induced to cardiomyocytes. The immunostaining of Caspase 3 in the differentiate cells. DNA was stained with Hoechst 33342. Scale bar, 50 μm (D) Blimp1/Stella-mfPSCs were transformed onto Matrigel coated dishes and cultured for another 4-5 days. The Blimp1⁺ (green) and Stella⁺ (blue) cells displayed in the PGCLCs aggregates from 2D-adapted mfPSCs at day 6. Scale bar, 100 μm.
